# DNA damage response signaling pathways and targets for radiotherapy sensitization in cancer

**DOI:** 10.1038/s41392-020-0150-x

**Published:** 2020-05-01

**Authors:** Rui-Xue Huang, Ping-Kun Zhou

**Affiliations:** 1 Department of Occupational and Environmental Health, Xiangya School of Public Health, Central South University, 410078 Changsha, People’s Republic of China; 2 Department of Radiation Biology, Beijing Key Laboratory for Radiobiology, Beijing Institute of Radiation Medicine, AMMS, 100850 Beijing, People’s Republic of China; 3 Institute for Chemical Carcinogenesis, State Key Laboratory of Respiratory, Guangzhou Medical University, 511436 Guangzhou, People’s Republic of China

**Keywords:** Cell biology, Genetics research

## Abstract

Radiotherapy is one of the most common countermeasures for treating a wide range of tumors. However, the radioresistance of cancer cells is still a major limitation for radiotherapy applications. Efforts are continuously ongoing to explore sensitizing targets and develop radiosensitizers for improving the outcomes of radiotherapy. DNA double-strand breaks are the most lethal lesions induced by ionizing radiation and can trigger a series of cellular DNA damage responses (DDRs), including those helping cells recover from radiation injuries, such as the activation of DNA damage sensing and early transduction pathways, cell cycle arrest, and DNA repair. Obviously, these protective DDRs confer tumor radioresistance. Targeting DDR signaling pathways has become an attractive strategy for overcoming tumor radioresistance, and some important advances and breakthroughs have already been achieved in recent years. On the basis of comprehensively reviewing the DDR signal pathways, we provide an update on the novel and promising druggable targets emerging from DDR pathways that can be exploited for radiosensitization. We further discuss recent advances identified from preclinical studies, current clinical trials, and clinical application of chemical inhibitors targeting key DDR proteins, including DNA-PKcs (DNA-dependent protein kinase, catalytic subunit), ATM/ATR (ataxia–telangiectasia mutated and Rad3-related), the MRN (MRE11-RAD50-NBS1) complex, the PARP (poly[ADP-ribose] polymerase) family, MDC1, Wee1, LIG4 (ligase IV), CDK1, BRCA1 (BRCA1 C terminal), CHK1, and HIF-1 (hypoxia-inducible factor-1). Challenges for ionizing radiation-induced signal transduction and targeted therapy are also discussed based on recent achievements in the biological field of radiotherapy.

## Introduction

The increasing prevalence of cancer worldwide is a major challenge to the improvement of quality and length of life. According to reports, 975,396 patients were newly diagnosed with cancer in 2012, and there were 358,392 deaths due to cancer in youth globally^1^. In 2015, the number of identified cancer cases increased to ~17.5 million, and the deaths from cancers increased to 8.7 million globally. Notably, from 2005 to 2015 (an 11-year span), the number of patients with cancer increased by 33%^2^. In 2016, ~9 million deaths were attributed to cancer, an increase of almost 18% over one decade^3^. Moreover, in the United States in 2017, there were 1,688,780 newly diagnosed cancer patients and almost 600,920 deaths due to cancer^4^. These numbers have increased rapidly annually, and in 2018, ~18.1 million newly diagnosed cancer patients and 9.6 million cancer deaths were reported worldwide^5^. In China, in 2014, there were 3.804 million newly diagnosed cancer patients and 2.296 million cancer deaths, and the statistical results showed that the crude incidence rate and the crude mortality rate were 278.07 per 100,000 people and 167.89 per 100,000 people, respectively^6^. Furthermore, it is estimated that by 2035, the number of annual cancer deaths will reach 14.5 million because worldwide cancer cases are expected to dramatically increase from 15 million at present to 24 million in the next 20 years^3^. Moreover, in parallel with the increasing rates of cancer diagnosis and death, the global burden of cancer has gradually increased over the past decade. Based on the Global Burden of Disease Cancer Collaboration announcement, 208.3 million disability-adjusted life-years (DALYs) were attributed to cancer globally in 2015. Lung cancer was the top cause of death among males, accounting for 25.9 million DALYs, while in females, breast cancer-attributable deaths were the top cause, accounting for 15.1 million DALYs^2^. Significant advances in the war against cancer have been achieved over the past decade. For instance, deaths from Hodgkin lymphoma declined significantly between 2005 and 2015 (−6.1%; 95% uncertainty interval: −10.6% to −1.3%), and other cancer deaths, such as deaths from esophageal cancer and stomach cancer, have also significantly decreased over the past decade^2^. Additionally, through large-action control of tobacco use and human papillomavirus vaccination in females, the burden of cancer in the female population has been substantially decreased in both economically developed and economically developing areas^7^. Although large-scale implementation of prevention and treatment methods has made these improvements possible, there is still a long way to go in the fight against cancer.

The management of cancer mainly involves surgery, radiotherapy, chemotherapy, and the rapidly evolving field immunotherapy^8^. The most commonly used cancer therapies over the past century include chemotherapy and radiotherapy methods^9–12^; among these, radiotherapy is widely and predominantly used prior to surgery and other treatment methods^13,14^. Radiotherapy is defined as the application of radiation for clinical cancer treatment, including external-beam radiation and local radioactive seed implants with the purpose of killing cancer cells or controlling cancer cell proliferation^15,16^. Radiotherapy is sometimes used alone, but at most times, it is applied in combination with other therapy strategies, such as surgery or oral medicine. Radiotherapy developed rapidly following the discoveries of X-rays by Roentgen, natural radioactivity by Becquerel, and radium by Curie over 125 years ago^17^. These three fundamental discoveries not only earned the discoverers Nobel Prizes but also founded the research field of radiology, as well as led to the establishment of radiotherapy techniques, such as external-beam radiotherapy with a long source surface distance and brachy therapy with a short-spacing surface space, which are commonly delivered through radium and X-rays^18^. Three countries, France, America, and Sweden, were the first to adopt radiation for gastric cancer and basal cell cancer treatment^19,20^. Over the past century, continuous technological improvements in radiotherapy have translated into better clinical practices, not only changing several fundamental concepts but also gradually changing clinical treatment guidelines. For instance, for a long time, radiation with a specific beam energy, such as telecobalt therapy, was applied in the clinic from 50–250 kV to 1.2 MeV, while the linear accelerator was between 6 and 20 MV; now, the computer revolution has made a three-dimensional (3D) approach in complex spaces a reality^21^. In addition, newly developed therapies based on high linear energy transfer (LET) particles, including protons and heavy ions such as carbon ions, are being used in cancer treatments^22–25^. However, with the increased usage of radiation not only in cancer treatment but also in medical examination globally, the adverse influence of radiation on the human body has attracted much attention in the public and scientific community, including the subsequent secondary cancer risks and damage to normal tissues after radiotherapy^26^. Indeed, advances in radiation and radiotherapy are contributing substantially to winning the battle against cancer^27–29^. Currently, ~50% of cancer patients are subjected to radiotherapy. The combination of radiotherapy, surgery, and other medical treatments has contributed to almost 50% of cancer patients having a long-term survival opportunity. A study performed in Australia reported that among newly diagnosed patients, almost 52% of patients were subjected to radiotherapy, and more than 23% of patients required multiple treatments for a better prognosis^30^. In China, more than 50% of clinically treated cancer patients have received radiotherapy, which contributed to cure in more than 40% of patients^31,32^. To standardize quality control, a set of basic guidelines for radiotherapy have been developed, in accordance with the relevant national laws and regulations and referring to relevant international guidelines; These guidelines were announced at the 14th National Congress of Radiation Oncology (CSTRO; available at https://cstro2017.medmeeting.org/cn) meeting^32^. Moreover, radiotherapy is a conservative treatment with the capacity to affect cancer without body image alteration. Most importantly, radiotherapy is very cost effective. The data released by the International Atomic Energy Agency suggest that radiotherapy is the most economical treatment measure overall, accounting for ~5% of the all-in expenses of patient care for cancers^33^. Most experts hold that treating cancer using radiation technology is essential and critical for not only saving thousands of cancer patient lives but also saving economic costs in cancer patients; thus, access to radiotherapy should be available globally in the near future ^34^.

However, radiotherapy is typically accompanied by the unavoidable development of cancer cell resistance to radiation exposure^35,36^. Radiotherapy resistance (RR), defined as a reduction in the effectiveness of antitumor therapy^37^, is a major obstacle in cancer treatment. RR either arises within cancer cells when cancer cell genes or phenotypes are altered in response to radiation exposure or is due to the cancer microenvironment protecting cancer cells against the treatment. The former is referred to as intrinsic resistance, while the latter is referred to as extrinsic resistance^38^. RR leads to cancer relapse, poor treatment response, poor prognosis, decreased quality of life, and increased disease treatment burden. Furthermore, RR induces damage to cancer-adjacent normal tissues, disrupting the physiological and biochemical functions of normal tissue, resulting in symptoms, including radiation-related diarrhea, rectal bleeding, and radiation dermatitis^39–41^, as well as an increased risk of subsequent secondary cancer^26,42–44^ or chronic noncommunicable diseases including type II diabetes^45,46^ or cardiovascular diseases^47^. Over the past century, to remove the barrier of RR, many studies have been carried out to investigate RR-related regulatory genes, molecules, and signaling pathways to uncover the underlying mechanisms of RR and to develop radiation sensitizers^48,49^. Currently, two large bottlenecks for successfully improving radiation resistance are as follows: (1) identifying the master regulator of the development and progression of RR and (2) determining how the master regulator can serve as a potential target to overcome RR. In this review, we discuss the promising targets of signaling pathways that can be proposed for cancer radiosensitization and may be translated into clinical radiotherapy targets.

## DSBs are a major pattern of RIDD

There are many factors associated with increased RR in cancer cells. These factors include but are not limited to the following: the local cancer microenvironment, membrane signaling sensors, and the patient immune system, gut microbial community^50^, nutritional status^51^, and mental health status^52^. However, among the reported and discovered factors, DNA damage is a primary and intrinsic factor and the most crucial operator in the response to radiation exposure and the orchestration of the subsequent cascade of DNA repair response signaling pathways to control cancer cell cycle arrest and cell fate, i.e., death or survival^53^. In other words, the ability of radiation to control cancer predominantly depends on radiation-induced DNA damage (RIDD)^53^; as a result, the DNA damage response (DDR) of tumor cells and the ability of tumor cells to repair DNA damage are essential in determining the outcome of cancer cells.

The genomic integrity of cells is extremely important for cell growth as well as successful transmission of genetic information to the next generation^54^, However, many types of external or internal genotoxic insults challenge DNA integrity^55^, forcing the host to evolve and develop compensatory changes to combat DNA damage and maintain genomic integrity^56^ via several independent or complementary DNA repair pathways, allowing for a fail-safe mechanism whereby the disruption of one pathway will be compensated for by another pathway^57^. During cancer cell evolution, multiple comprehensive molecular signaling pathways have been developed to face the challenge of radiation stress, and this ability to evolve can contribute to increased cancer cell RR, leading to radiotherapy failure. Moreover, during the process of developing RR, a percentage of cells in tumor tissues not only acquire higher RR but also become more aggressive and are prone to lymph node and distant metastasis^58^. Thus, enhancement of the cancer response to radiation through DNA damage pathways has been a focus of radiotherapy studies for the past few decades ^59–61^.

**Fig. 1:**
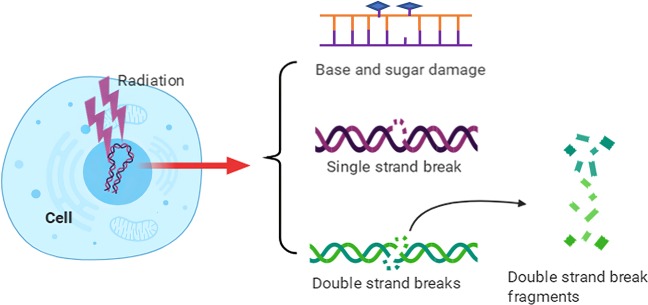
DNA damage induced by ionizing radiation. The major types of DNA damage induced by IR include base and sugar damage, single-strand breaks, double-strand breaks, clustered DNA damage, and covalent intrastrand or interstrand crosslinking

Typically, exposure to ionizing radiation (IR) is often suggested as a treatment for preventing cancer cell proliferation. There are various applications depending on the IR type, such as electromagnetic waves or particles. Currently, several techniques and standard values are well accepted for IR applications; for example, LET is usually performed at between 1 and 10 keV/µm using sources such as X-rays, γ-rays, protons, or carbon ions, while very high values, usually beyond >100 keV/µm, are found in some forms of space radiation^62^. As reported previously, once the radiation track deposits its energy in the DNA molecules of cancer cells, a fraction of the DNA damage sites will have two or more damages formed within one or two helical turns of DNA^63,64^. The common DNA damage pattern comprises base and sugar damage, crosslinks, single-strand breaks (SSBs) and double-strand breaks (DSBs), as shown in Fig. 1. Compared to those of SSBs, the patterns of DSBs are more intricate and include simple and complex types, as well as multiple physical characteristics not only referring to the break but also to the kinetics of the ability to repair the break^65,66^ In terms of simple DSBs, two-ended breaks of DNA may occur as a direct result of radiation, and these have rapid kinetic repair. Complex DNA DSBs, namely, “clustered DNA damage,” are a hallmark of IR and locally consist of more than two instances of oxidative base damage, basic sites, or SSBs around a DSB^63,67,68^. Compared to simple breaks, complex DSBs are more slowly and inefficiently repaired, resulting in genomic instability^69–71^. Indeed, the radiation exposure response to DNA damage may vary based on IR type. For instance, high-LET radiation, involving methods such as heavy ion and proton radiation, may preferentially induce clustered damage, a signature of IR; in contrast, isolated, endogenously induced lesions result in a homogeneous distribution^67,72^. Following irradiation with X-rays or γ-rays, clustered DNA lesions are often 3–4 times more abundant than single-strand damage^67,73^. Actually, the number of DSBs resulting from high-LET irradiation is much more than that resulting from low-LET irradiation. There is evidence that high-LET irradiation causes almost 500 DSBs/μm^3^ track volume^74^. Using 3D-structured illumination microscopy, Hagiwara et al.^75^ revealed that clustered DSBs could be formed in a space of 1 μm^3^ by high-LET irradiation; moreover, once these clustered DSBs occur, a higher risk of chromosomal rearrangement and lethality will subsequently develop. More efficient induction of complex clustered DNA damage is a major factor contributing to the higher relative biological effectiveness of heavy ions.

Moreover, once a complex DSB forms, the repair occurs slowly, and chromosomal aberrations can cause cell death or delayed mitosis without further repair^73,76^. Because IR induces genetic instability, RR is expected if cancer cells survive following treatment with IR. Hence, how cellular sensors respond to radiation and how early signal transducers work after IR will be reviewed in the next section based on recently reported evidence to understand the mechanisms of DNA damage signals more deeply and clearly.

## Cellular DNA damage sensors and early signal transducers in response to IR

As a signal, DNA damage activates a series of biochemical reactions in response to IR insult, triggering a variety of cellular responses. Nevertheless, the key questions are how the DNA damage signal is sensed and recognized and how the cascade signaling of downstream biochemical reactions is triggered. DNA damage sensors and early signal transducers thus play essential roles in recognizing DNA damage^77,78^. The ideal DNA damage sensors are the first proteins to contact DNA damage sites, identifying damage signals and triggering cell signaling transduction^79–81^. Moreover, DNA damage sensors also have the ability to recruit DDR proteins to sites of DNA damage^82,83^. Signal transducers often play roles as functional partners of DNA damage sensors^83,84^. As DNA damage sensors and signal transducers usually coexist, it is difficult to classify them. However, signal transducers have kinase activity, transducing the DNA damage chemical signal to induce biochemical modification reactions and triggering the activity of downstream effectors ^85^.

The first DSB sensor identified from fission yeast was Rad24p by Ford et al. in 1994^86^; this sensor is required for DNA damage checkpoint (DDC) activation and is essential for cell proliferation^87^. In *Schizosaccharomyces pombe*, Rad24p is required for some essential functions, as double deletion of its encoding gene is lethal^87,88^. As a DNA damage sensor, Rad24p is often considered to be the primary DNA damage responder, forming a complex with Ddc1p and Mec3p and triggering cell cycle arrest after DNA damage^89^. Furthermore, previous studies have revealed that Rad24p associates with Rfc2p-Rfc5p to form replication factor C, functioning in DNA replication or repair and DDC pathways^90^. In Rad24p-containing compounds, the Rad24p-Rfc2p or Rad24p-Rfc5p complexes can recruit the Rad24p-Ddc1p-Mec3p complex to create a “workshop” similar to the reaction machinery, triggering downstream kinases or effectors such as Rad53p^89,91–93^. The 14-3-3 isoforms are the mammalian homologs of fission yeast Rad24, functioning in DDC as well as in cell cycle control^94^. Following the discovery of Rad24p as the DSB sensor, Mec1p and Rad26p were subsequently identified as DNA damage sensors as well^89,95^. Mec1p has been considered to be the regulator of Ddc1p phosphorylation, a protein responsible for the yeast DDC^89^. Mec1p and Rad26p, from budding yeast and fission yeast, respectively^96^, have the characteristics of DNA damage sensors, activating phosphatidylinositol proteins^97^. The phosphorylation substrates of Mec1p and Rad26p include Ddc1p and Rad9p, another two DNA damage sensors^98–100^. In general, DNA response mechanisms have been extremely conserved during evolution within both yeast and mammalian cells. Although no effective or idealized DNA damage sensors have been confirmed in mammalian cells, several important molecules were recognized to be associated with DNA damage sensors and, importantly, to mediate and trigger IR-induced DSB signaling responses.

### γH2AX

As reported by Siddiqui et al.^101^, H2AX can respond to DSBs in a phosphorylation pattern at a very early time. H2AX is a variant of the core histone protein H2A; upon DNA DSB occurrence, H2AX is phosphorylated at the S139 site, which forms γH2AX foci. Furthermore, in their review, Siddiqui et al.^101^ mentioned that γH2AX persisted after exposure to IR under treatment with various radiosensitizing drugs, indicating that this sensor could be used to monitor cancer therapy and to tailor cancer treatments. Using anti-γH2AX monoclonal antibodies and immunofluorescence hybridization techniques to visualize γH2AX localization at sites of DNA damage^102,103^, even with a very low dose of IR exposure, γH2AX foci can be visualized as well, but once the DNA damage is repaired, the foci are eliminated^104,105^. Kuo and Yang^106^ suggested that γH2AX foci represent DSBs in a 1:1 ratio and can be used as a biomarker for DNA damage^106^. Specifically, the disappearance of γH2AX typically occurs earlier than that of other IR exposure response proteins. Moreover, γH2AX can act as a platform to recruit other DNA repair proteins, such as BRCA1 (BRCA1 C terminal)^107^, 53BP1 (p53-binding protein 1),^108^, MDC1^109^, and Rad51^110^. Zhang et al.^111^ reported that glioma stem cells exhibited RR with increased γH2AX-positive cell rates after 6 Gy radiation due to upregulation of the long noncoding RNA (lncRNA) PCAT1. Katagi et al.^112^ evaluated the effects of histone demethylase inhibition on genes associated with DSB repair in diffuse intrinsic pontine glioma cells and found that the expression of DSB repair genes was significantly reduced, but the level of γH2AX increased and was sustained at a high level. Overall, γH2AX was considered to match the characteristics of a DNA damage sensor more closely than the expression of DSB repair genes. Currently, γH2AX is widely used as a marker to detect radiation-induced DSB repair by immunofluorescent staining of foci or immunocytochemistry^113,114^.

### Nbs1/hMre11/hRad50 complex

**Fig. 2:**
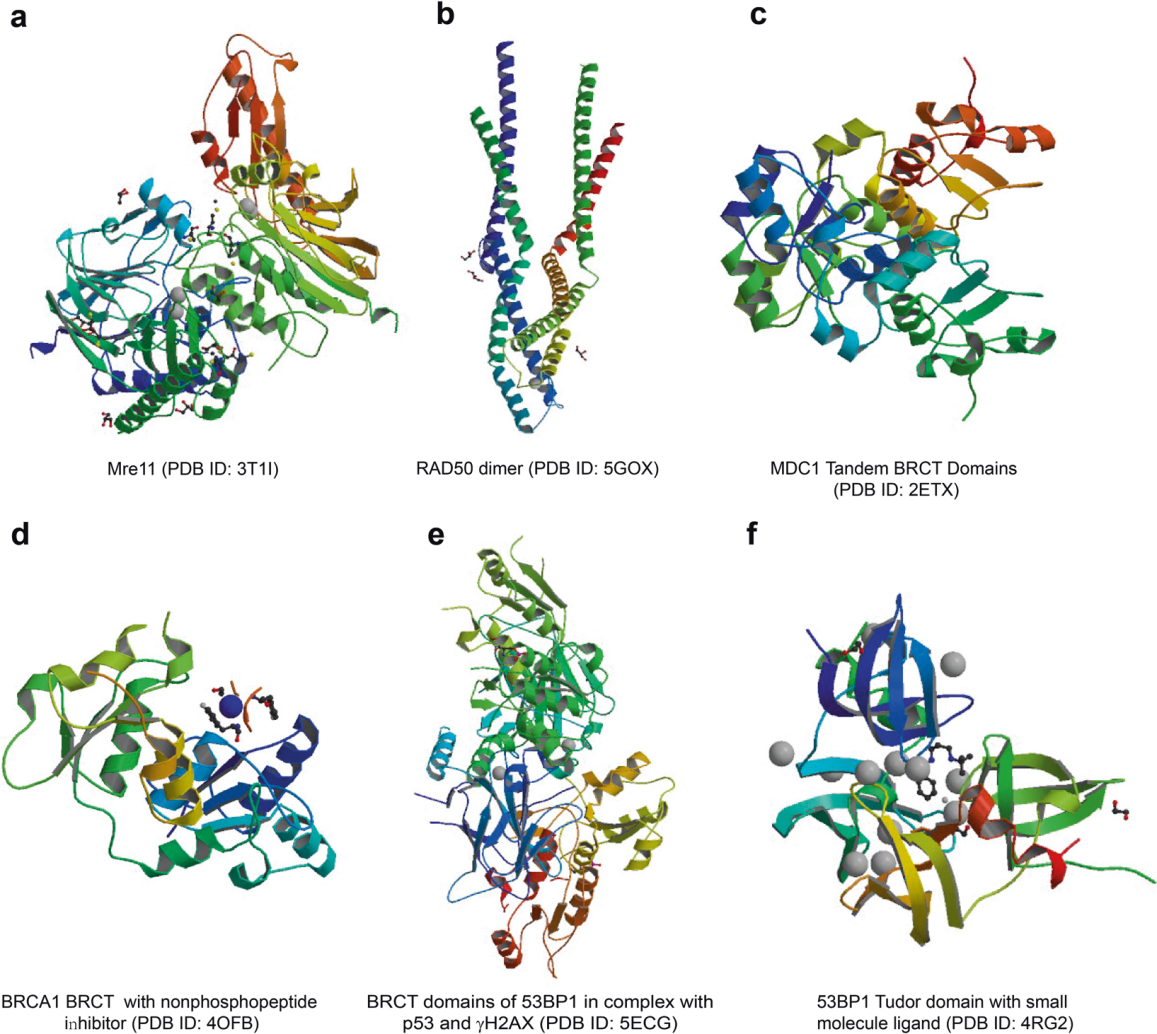
Structures of major DNA damage signal sensors, their main functional domains and their interactions with their partners. The data are from the RCSB database (https://www.rcsb.org/)

The MRN (Mre11-Rad50-Nbs1) complex, formed by Nbs1, hMre11, and hRad50, was first reported by Carney et al. in 1998^115^. According to this report, the MRN complex is responsible for linking DSB repair with cell cycle checkpoint functions. Habraken et al.^116^ suggested that the Nbs1/hMre11/hRad50 complex plays an important role in DNA DSB sensing and the signal transduction initiated by X-ray radiation. Similarly, a study by Kobayashi^117^ demonstrated that one of the roles of this Nbs1/hMre11/hRad50 complex is to recruit activated ataxia–telangiectasia mutated (ATM) to DNA damage locations, showing that it has the ability to recognize DNA damage initially. Moreover, according to Tauchi et al.^118^, the hMre11 binding region is necessary for both nuclear localization of the Nbs1/hMre11/hRad50 complex and for cellular radiation resistance; meanwhile, the fork head-associated domain of Nbs1 regulates nuclear foci formation of the multiple proteins in the complex. The hMre11 structure is comprised of an N-terminal core domain containing the nuclease and capping domains and a C-terminal domain containing the DNA-binding and glycine-arginine-rich motif (Fig. 2a). Deletion of the α2-β3 loop (AA84–119) in the hMre11 core structure prevented the formation of a stable hMre11 core dimer and inhibited Nbs1-binding activity^119^. The structure of the MRN complex contains two major dimerization interfaces that link Mre11, Rad50, and Nbs1 in DNA damage sensing and signaling. One dimerization interface is within the globular domain and involves Rad50 and Mre11. The second is the zinc hook situated distal to the globular domain separated by the antiparallel coiled-coil domains of Rad50. The two parallel coiled-coil domains of RAD50 proximal to the hook form a rod shape (Fig. 2b), which is crucial for stabilizing the interaction of Rad50 protomers within the dimeric assembly^120^. In the presence of Mn^2+^ ions, the hMre11 core shows exonuclease and endonuclease activities for 30–50 base spans. Structure and biochemistry analyses indicate that many tumorigenic mutations of hMrell are primarily associated with its Nbs1 binding and partly with its nuclease activities^119^. A study indicated that heterozygous p.l171V mutation in the *Nbs1* gene was found in Korean patients with high-risk breast cancer^121^. In addition, a persistent increase in radiation-induced Nbs1 foci formation was accompanied by an increased frequency of spontaneous chromosome aberrations^122^. Another in vitro study indicated that heterozygosity of *Nbn*, the murine homolog of human *Nbs1*, contributes to mouse susceptibility to IR-induced tumorigenesis^123^. Della-Maria et al.^124^ further identified a novel mechanism in which the Nbs1/hMre11/hRad50 complex interacts with the DNA ligase III α/XRCC1 complex, which is linked with the nonhomologous end-joining (NHEJ) pathway in cancer cells following IR radiation. Ho et al.^125^ demonstrated that overexpression of the Nbs1/hMre11/hRad50 complex in rectal cancer was associated with RR and poor prognosis. Collectively, although Nbs1 is the phosphorylation target of ATM, the Nbs1/hMre11/hRad50 complex localizes upstream of ATM in the DDR, acting as a sensor.

### Ku (Ku70/Ku80 heterodimer)

Once DSBs occur under IR stress, the primary repair pathways are triggered through two classical pathways, NHEJ and homologous recombination (HR). The NHEJ pathway is triggered via Ku, also known as the Ku70/Ku80 heterodimer, which is preferentially recognizes DSBs^126^. Depletion of the deubiquitylating enzyme UCHL3 resulted in the reduced chromatin-binding and IR-induced foci (IRIF) formation of Ku80 after DSB occurrence, moderately sensitizing cancer cells to IR^127^. A recent study by Pucci et al.^128^ reported nuclear localization of Ku in advanced rectal cancer patients with superior sensitivity to radiotherapy. However, in nonresponder patients, Ku70 was found to move from the nucleus to the cytoplasm, and strikingly, deregulation of Ku70/80 and the Ku70 partner clusterin was extensively associated with RR. Another study found that IR induces the accumulation of autophagosomes, and the radiosensitizing effect of autophagy-related BECN1 deficiency may result from the disruption of nuclear translocation and Ku protein activity, leading to the attenuation of DSB repair in malignant glioma. Similar to other DNA damage sensors, Ku includes a pocket structure. Once DNA damage occurs, Ku can bind to the DNA damage site and immediately embed the DNA break terminus into this pocket^129^. In a clinical study, the B cells of some B cell chronic lymphocytic leukemia patients were resistant to IR-induced apoptosis; when the B cell chronic lymphocytic leukemia cell subset was treated with radiation, the DNA end-binding ability of Ku was significantly increased by two- or three-fold in the radiation-resistant cell subset compared with that in the radiation-sensitive cell subset ^130^.

### MDC1 and 53BP1

DDR alterations are a major cause of cancer cell resistance to radiotherapy^131^. Both proteins, one named mediator of MDC1 and another known as 53BP1, are also associated with the signaling of DNA DSBs^131^. MDC1 is a major modular phosphoprotein scaffold that plays an important role in the DDR process. The BRCT domains are crucial modules that mediate the protein–protein interaction in DNA damage sensing and DDR signaling and have been found in a number of DDR proteins, such as MDC1, 53BP1, BRCA1, and Nbs1. In addition to their conserved phosphopeptide recognition and binding functions, BRCT domains are also implicated in phosphorylation-independent protein interactions, poly(ADP-ribose) (PAR) binding and DNA binding^132^. The architecture of BRCT domains is variable, ranging from a single module to tandem BRCT repeats. Figure 2c–e displays the BRCT domain structures of MDC1, BRCA1, and 53BP1, respectively. Following induction of DNA DSBs, MDC1 is anchored to damaged sites through interaction of its BRCT repeat domain with the tail of γH2AX. Moreover, MDC1 often performs its roles in accompaniment with 53BP1; that is, it can make 53BP1 move to foci under the control of MDC1^131,133^. The function of 53BP1 in DDR is dependent on its recruitment to the damaged site through 53BP1 tandem Tudor domain-mediated recognition of methylated histone H4 (H4K20me2) and ubiquitinated histone H2A (H2AK15ub). 53BP1 binds with the DSB marker H2AX-pS139 through its BRCT_2_ domain in vitro and in cells (Fig. 2e), which is necessary for the recruitment of pATM to the damage site^134^. The Tudor domain of 53BP1 (Fig. 2f) also plays a critical role in the DDR through interactions with BRCT domains. As a 53BP1 regulator, the Tudor-interacting repair regulator (TIRR) directly binds to the 53BP1 Tudor domain and blocks the H4K20me2 binding surface. High-resolution structural analysis shows that the N-terminal region and the L8 loop of TIRR form an extensive binding interface with three loops of the 53BP1 Tudor domain^135^. TIRR masks the binding surface of H4K20me2. In colorectal cancer cells, following radiation, coimmunoprecipitation analyses showed that Ku70, γH2AX, and MDC1 were colocalized in nuclear foci^136^. Cairns et al.^137^ reported that MDC1 could be regulated by Bora. Another study showed that Bora could be phosphorylated by MDC1, leading to abolishment of irradiation-induced MDC1 foci formation, and downregulation of Bora increased the resistance to IR, likely due to a faster rate of DSB repair. A clinical study conducted by Cirauqui et al.^138^ showed that patients with head and neck cancer with low 53BP1 expression levels treated with radiotherapy had a higher complete response as well as a higher survival time than patients with high 53BP1. Generally, DNA damage induced by IR recruits MDC1 to sites of damage within ~1 min post irradiation, providing a γH2AX-dependent interaction platform for recruiting other DNA damage repair proteins, such as ATM and Nbs1, and the glycolytic enzyme PFKFB3.

### BRCA1 and BRCA2

BRCA1 and BRCA2 are clinically correlated with hereditary breast and ovarian cancer. For *BRCA*1 mutation carriers, the relative risk of breast cancer is 1.19 (95% confidence interval [CI]: 1.02, 1.39), while for *BRCA*2 mutation carriers, the relative risk of breast cancer is 1.25 (95% CI: 1.01, 1.55)^139^. In prostate cancer patients with resistance to prostate-specific membrane antigen-targeting α-radiation therapy, *BRCA1* and *BRCA2* genes were deleted, and several variants of *BRCA1* were detected^140^. BRCA1 consists of several domains, including an N-terminal region carrying the zinc-binding finger domain RING and two phosphopeptide-binding BRCT domains^141,142^. Similarly, there are also a few domains for BRCA2, that is, the transcriptional activation domain is located at the N terminus, and the DNA-binding domain is located close to the C-terminal region. Other regions include a conserved helical domain, three oligonucleotide binding folds, and a tower domain^141,143^. BRCA1 and BRCA2 play a crucial role in the repair of DSBs in the HR pathway^144^. After exposure to radiation, the BRCA1-RAP80-Abraxas complex binds ubiquitinated histone in response to DNA damage^145–147^. A recent report showed that BRCA1 could recruit CSB, a member of the SWI2 family, and MRN to form a complex at the late phase of S/G2. This interaction between BRCA1, CSB, and MRN is responsible for MRN-mediated DNA end resection^148^. In addition, the BRCA1-PALB2 interaction dictates the choice between HR and single-strand annealing^149^ and is associated with RR. These important roles of BRCA1/2 have suggested them as attractive, valuable, and sensitive diagnostic biomarkers in the prediction of radiotherapy outcomes ^34^.

The above discussion is associated with the progress of DDR-associated proteins; notably, with an increasing number of in-depth studies, some novel response proteins have been reported. A recent study found that a novel DDR was triggered by MT1-MMP (membrane-tethered matrix metalloproteinase)-integrin β1. This study indicated that suppression of MT1-MMP would improve breast cancer cell resistance to IR therapy ^150^.

**Fig. 3:**
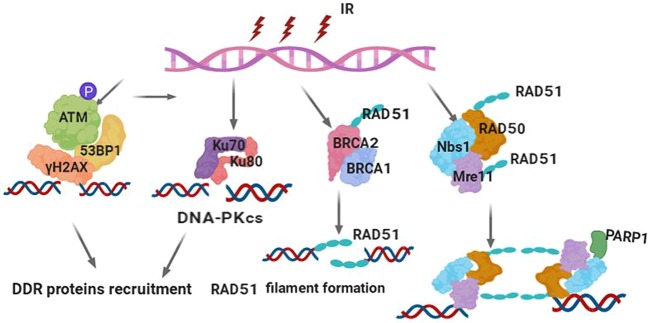
Damage sensors and their functional complexes in response to DNA double-strand breaks. (1) Upon DSB occurrence, the core histone protein variant H2AX is instantaneously phosphorylated on its S139 position to form γH2AX foci, which can be detected at the DSB site. γH2AX provides a platform to recruit DDR proteins, such as 53BP1, MDC1, and ATM, to DSBs to initiate DDR signal transduction. (2) DNA-dependent protein kinase (DNA-PK), composed of Ku70, Ku80, and the catalytic subunit DNA-PKcs, is a classical DSB-sensing and -binding complex. DSB binding by DNA-PK protects the broken DNA end from degradation by endogenous nucleases; on the other hand, it recruits and activates the downstream components in the NHEJ pathway of DSB repair. (3) BRCA1 and BRCA2 are key proteins involved in DSB binding and initiating the HR pathway and later repair processing. BRCA2 directly recruits RAD51 to sites of DNA damage through interaction with conserved BRCT motifs to stabilize the RAD51 nucleoprotein filament on the ssDNA end of DSBs. Following end resection of the DSBs, BRCA1 activates RAD51 to promote gene conversion of homologous recombination. (4) The MRN complex (Mre11-Rad50-Nbs1) is the primary sensor of DSBs and localizes to damage sites to initiate end resection and HR processing. The MRN complex also promotes the recruitment and activation of ATM and PARP-1. PARP-1 produces poly(ADP-ribose) polymers and extends DNA damage signaling

In brief, it is well known that IR-induced DSBs are the most deleterious form of DNA damage, leading to cell death and viable chromosomal rearrangements. As a result, cells have evolved an efficient and rapid DDR to maintain genomic integrity. DNA damage sensors are response proteins that can detect DNA damage; sensor proteins can also recruit transducer proteins to provide signals to enzymes to respond to the break. To date, a series of DNA damage sensor proteins have been identified through numerous studies, including γH2AX, 53BP1, Nbs1, BRCA1/2, and Ku. These DNA damage sensors commonly have the following characteristics: (i) they localize to the sites of DSBs within a few seconds or minutes after IR exposure, forming microscopically visible nuclear domains referred to as IRIF; (ii) sensor proteins can modify the adjacent damage sites by methods such as phosphorylation of γH2AX; (iii) sensor proteins can recruit other proteins to sites of damage to form protein complexes such as the Nbs1/hMre11/hRad50 complex^151^; and (iv) these DNA damage sensors can also regulate each other. For instance, MDC1 expression was induced by radiation, and the overexpression of MDC1 could activate Nbs1 activity in the presence of DNA damage repair^152^. These sensors can also be regulated by upstream or downstream proteins. For instance, the human demethylase JMJD1C was stabilized by interaction with RNF8 and recruitment of RAP80-BRCA1, and MDC1 was demethylated at Lys45 through JMJD1C binding to RNF8 and MDC1, promoting cancer cell sensitivity to IR^153^. Reichert et al.^154^ reported that following exposure to radiation, a direct relationship between MDC1, γH2AX, and 53BP1 was identified, and higher amounts of DNA breaks were associated with an increased level of γH2AX/53BP1 foci post irradiation. Thus, it is suggested that identification of these sensors after the occurrence of DSBs under IR exposure may be a predictive biomarker in determining radiotherapy outcomes among patients with cancer. γH2AX is a typical example of a marker that has been translated from bench to bedside, and it has been employed in the clinic as a predictive biomarker for radiotherapy sensitivity in some kinds of cancers^155^. Table 1 presents several primary DNA damage sensors along with their roles and characteristics. Figure 2 illustrates the structures of these sensor proteins or their main functional domains and interacting partners. Figure 3 illustrates the regulation of DNA damage sensors following IR exposure in terms of the common DSB sensors and early signal transducers. Based on the above discussion of the roles and characteristics of DNA damage sensors, these sensors could be used as biomarkers to detect or evaluate DNA damage induced by IR in routine clinical use to determine optimal radiation dosing or as future targets for overcoming RR ^156^.

**Table 1:** Summary of a few main DNA damage sensors induced by IR (human versions are shown)

	Length	Subcellular location	Interaction partners	Mutations^a^
γH2AX	143	Nucleus^436^, chromosome^437^	Several other proteins^436,438,439^	141 (Q to N)^440^
Nbs1	754	Nucleus^441^, telomere^442^, chromosome^441^	MCM9^441^; BRCA1, MSH2, MSH6, MLH1, ATM, BLM, RAD50, MRE11, and NBN^443^	28 (R to A); 45 (H to A); 136 (G to E)^444^
Mre11	708	Nucleus, telomere, chromosome^441^	MCM9^441,445^	104 (S to C) in cancer^446^
Rad50	1312	Nucleus, telomere, chromosome^441^	MCM8 and MCM9^441^; BRCA1^447^; MSH2, MSH6, MLH1, ATM, BLM, RAD50, MRE11, and NBN^443^	94 (I to L), 127 (V to I); 191 (T to I), 193 (R to W), 224 (R to H), 315 (V to L), 469 (G to A)^448^
MDC1	2089	Nucleus, chromosome^449^	MRE11, RAD50, and NBN; CHEK2, the BRCA1-BARD1 complex, SMC1A and TP53BP1, ATM and FANCD2^450,451^	58 (R to A) and 1840 (K to R)^451^
53BP1	1972	Nucleus^452^, chromosome^453^	p53/TP53^454^; H2AFX^438^; CHEK2^455^; RIF1^456^; PAXIP1, IFI202A, and SHLD2^457^	6, 13, 25, 29, 105, and 166 (S to A)
BRCA1	1863	Nucleus^458^, cytoplasm^459^	BARD1, UIMC1/RAP80, ABRAXAS1, BRCC3/BRCC36, BABAM2, and BABAM1/NBA1^460,461^; RBBP8^462^; CHEK1, CHEK2, BAP1, BRCC3, AURKA, UBXN1, and PCLAF^463^; H2AFX^436^	26 (I to A)^464^; 308 (S to N)^465^; 1143 (S to A) and 1280 (S to A)^466^; 1692 (D to N) and 1749 (P to R)^467^

^a^Available from https://www.uniprot.org/

## Signaling pathways of DDR and repair

### IR-induced DNA damage repair

**Fig. 4:**
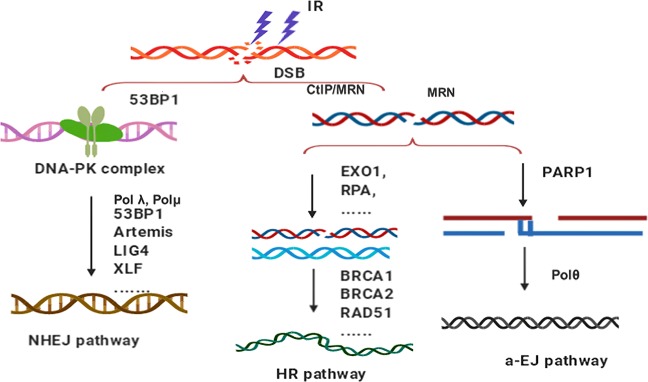
The pathways of DNA double-strand break repair. The nonhomologous end-joining (NHEJ) pathway is an error-prone repair pathway that functions through the cell cycle. The homologous recombination pathway is an error-free repair pathway that requires intact homologous DNA as a repair template and is active in the later S and G2 phases. The alternative end-joining (a-EJ) pathway, which repairs DNA double-strand breaks (DSBs), is initiated by end resection that generates 3′ single strand

IR kills cancer cells via the induction of DSBs in cancer cell genomic DNA, resulting in genomic instability, apoptosis, cell cycle checkpoint alteration, or postmitotic death. During IR treatment, cancer cells evolve personalized DNA damage repair mechanisms against IR insults for survival^66^. The induction of DNA mechanisms required to realize the effects of IR has been referred to as “hormesis”^157^. It has been reported that three different primary pathways evolved to process DSB repair: the HR-based pathway, NHEJ, and alternative end joining (Fig. 4). The goal of these repair pathways is to handle different forms of DNA lesions, eventually achieving DSB removal and maintaining genomic integrity ^158^.

Understanding the underlying mechanisms by which DNA damage is repaired in cancer cells post IR treatment would facilitate overcoming RR^159^. For instance, eurycomalactone, an active quassinoid isolated from *Eurycoma longifolia Jack*, markedly delayed the repair of radiation-induced DSBs in non-small-cell lung cancer (NSCLC) cells^160^. Koval et al.^157^ reported that a protective system could be activated among cancer cells in response to IR, leading to increased resistance to subsequent exposure to IR, and moreover, chronic exposure to γ-rays increased the expression of the *mus210*, *mus219*, and *mus309* genes, even after 56 days, in Canton-S flies. Although the development of genome-wide sequencing techniques has allowed scientists to identify the molecular mechanisms of the radiation-induced adaptive response, including the Notch, tumor growth factor-β, mammalian target of rapamycin, and Wnt signaling pathways, the detailed mechanism of cancer cell defense in IR-induced hormesis remains unclear.

### DNA DSB repair pathways

In the history of studying the DSB repair pathway, the HR pathway was the first to be discovered^161^. The HR pathway was named due to the close vicinity of homologous strands during mitosis. HR is specifically triggered in cells in the later S and G2/M phases^162^. In the 1980s, the second DSB repair pathway, the DNA end-joining pathway, was discovered. In contrast to HR, NHEJ is triggered in the G0/G1 phase as well as G2/M^163^. Nevertheless, NHEJ is supposed to be predominant in mammalian cells compared with microorganisms^164,165^. Since the term homologous was used previously in the HR pathway by radiobiological community, the second discovered pathway was defined as NHEJ^166,167^. However, some radiobiologists disagree with the naming approach and have suggested the existence of other DSB repair pathways because studies have revealed that in cancer cells with extreme radiotherapy sensitivity, both the HR and NHEJ pathways exist, suggesting that other repair pathways may also be functioning^168^.

Many studies have indicated that HR is essential for accessing the redundancy of genetic information that exists in the form of sister chromatids or homologous chromosomes when both strands of the DNA double helix are compromised^169^. When a chromosome is insulted by IR exposure, the DDR cooperates with cellular signaling pathways to maintain genomic stability and ensures cell survival^170,171^. As shown in Fig. 4, for the HR pathway, the DSB is resected from 5′ to 3′ on one strand of the DSB end, creating terminal 3′-OH single-strand DNA (ssDNA) tails^169^. In other words, during the process of HR repair, a homologous sequence is needed as the template^172^, aiming to restore HR accurately. Moreover, both one- or two-ended DSBs can be repaired through the HR pathway, and in particular, messy DNA breaks with covalently attached proteins can be repaired through HR^169,173^. Compared to NHEJ, HR is more complicated, involving numerous enzymes and proteins, but is more accurate and error free^174^. In summary, HR is slow, requires a template, is highly accurate, is only initiated at the later S and G2 phases, can repair both one- and two-ended breaks, and can repair protein-blocked ends^175,176^. The HR pathway for DSB repair has also been used as a genome editing tool. For instance, the clustered regularly interspaced short palindromic repeats (CRISPR)-Cas9 technique is now exploited in genome editing and is considered an incredible opportunity for curing genetic diseases^177,178^. Furthermore, the HR pathway is associated with RR. A recent study by Jin et al.^179^ found that *Deinococcus* shows high resistance to IR exposure due to a combination of passive and active defense mechanisms, such as self-repair of DNA damage through the HR pathway. Lopez Perez et al.^180^ reported that glioblastoma cancer cells exposed to carbon ions initiated the HR repair pathway with strong and long-lasting cell cycle delays, predominantly in G2, with a high rate of apoptosis. Many cancer cells aberrantly express the cancer/testes antigen HORMAD1. Knockout of HORMAD1 in cancer cells resulted in increased sensitivity to IR treatment, and the HR-mediated repair pathway targeting IR-induced DSBs was attenuated in HORMAD1-knockout cancer cells^181^. In addition, the cysteine protease cathepsin B contributes to RR by enhancing HR in glioblastoma^182^.

The basic mechanism of the CRISPR-Cas9 editing approach is to induce a site-specific DSB via bacteria, and the selection of the DSB repair pathway dictates the outcome of the editing^183^. That is, imprecise repair via the NHEJ pathway contributes to insertion or deletion mutations at the break sites; by contrast, repair via the HR pathway enables activation of the recombination machinery, which consequently deals with DNA segments or corrects pathogenic mutations^184,185^. The initiation of the HR pathway primarily occurs at the DNA break location, which function as ssDNA that can be used for locating a homologous dsDNA sequence. This sequence can then be used as a template for largely accurate repair. Meanwhile, extended DNA end resection contributes to nonligatable DNA breaks and hampers end joining; consequently, DNA end resection is dominant^186–188^. Hence, HR is initiated only when a repair template exists, which can limit the potential for illegitimate recombination. In general, misregulation of the HR repair pathway is critical for the generation of genome rearrangements in numerous cancers. It is important and necessary to further elucidate the details of HR, the roles of the relevant proteins, and how these proteins are regulated. We believe this will be an exciting direction in the future.

During the process of NHEJ, Ku first moves to and binds with DNA ends, and the binding shape is similar to a ring encircling the duplex DNA. This binding shape avoids DNA end degradation and recruits other proteins, such as DNA-dependent protein kinase, catalytic subunit (DNA-PKcs). After ligation of the broken DNA ends by the XRCC4-XLF complex and DNA ligase IV (LIG4), DNA-PKcs tethers with the Ku70/Ku80 ends^172^. NHEJ also plays an important role in cancer cell RR. Compared to HR, NHEJ generally has a rapid response, is template-independent, is often mutagenic, is cell cycle-independent, can only repair two-ended breaks, and cannot repair protein-blocked ends^189,190^. Bylicky et al.^191^ reported that increased expression of Ku70 may be a key factor for RR in normal human astrocyte cells; following X-ray radiation, the cells displayed a robust increase in the expression of NHEJ repair pathway-related enzymes within 15 min of radiation. Mu et al.^192^ reported that mangiferin induces sensitization of glioblastoma cells to radiotherapy via inhibition of the NHEJ repair pathway through regulation of various proteins, such as phosphorylation of ATM, 53BP1, and γH2AX. Wang et al.^193^ found that the lncRNA LINP1 facilitated DNA damage repair by decreasing the levels of cleaved caspase-3 and poly[ADP-ribose] polymerase (PARP) in response to IR and decreased the radiosensitivity of cervical cancer cells through the NHEJ pathway. These data show that the NHEJ repair pathway plays critical roles in controlling RR.

In cancer cells, both HR and NHEJ are important pathways for repairing DSBs caused by IR insult. For instance, the MEK1/2 (mitogen activated protein kinase kinase 1/2) inhibitor GSK212 mediates radiotherapy sensitivity by functionally repressing both HR and NHEJ, leading to delayed DNA repair and the persistent increased expression of γH2AX^194^. Thus, in clinical applications, upregulation of DNA repair pathways is recognized as a primary acquired mechanism through which cancer cells may become RR. Accordingly, radiotherapy sensitization strategies functioning via inhibition of IR-induced DNA repair and functional downregulation of the activity of both the HR and NHEJ pathways are expected to be applied clinically to control cancer. Figure 4 illustrates the repair pathways for DNA DSBs.

### Activation of cell cycle checkpoints

The cell cycle is essential for cell growth, proliferation, and reproduction. The cell cycle allows cellular components to be replicated and delivered to the next generation of cells^195^. The cell cycle is a complex process that involves a large number of regulatory proteins, including cyclin family proteins, cyclin-dependent kinases (CDKs), CDK inhibitors (CKIs), including Ink4 family members (p15, p16, p18, and p19) and Cip/Kip family members (p21, p27, and p57), CDC25 isoforms, p53 family proteins, and MDM2^196^. Over the past few decades, studies of the cell cycle have attracted extensive attention in the scientific community. Generally, in eukaryotes, the cell cycle can be divided into four phases, termed G1 (the first gap period), S (synthesis, the phase in which DNA is replicated), G2 (the second gap period), and M (mitosis)^197^. Cell cycle checkpoints, which define the end product of a molecular regulatory pathway or signaling cascade, ensure an ordered succession of cell cycle events, and when perturbed, lead to cell cycle arrest^198^; these checkpoints are critical for protecting cells from progressing into the next phase of the cell cycle before prior molecular events, such as DNA damage and spindle structure disruption, have been resolved^199^. If premature entry into the next cell cycle phase occurs without checkpoint review, catastrophic consequences or cell death may occur^200^. Cell cycle arrest is caused by depletion of some key proteins regulating this process. The cell cycle can be thought of as similar to a wheel, while the cell cycle checkpoints are the spokes of the wheel. The running of the wheel normally can maintain the doubling of cellular components and their accurate segregation into the next generation of cells. The spokes of the wheel are the regulators of the cell cycle and play an essential role in the function of checkpoints.

Cell cycle checkpoints are classified as DNA structure checkpoints (DSCs, or DDCs) and spindle assembly checkpoints (SACs). IR-induced DNA damage is one of the major triggers for the activation of a number of DNA structure checkpoints, leading to cell cycle arrest at various points in G1, S, and G2/M^201,202^. The SAC functions in the mitotic phase. In summary, IR-induced DSBs are a key signal for activation of cell cycle arrest at several cell cycle stages: termed G1/S arrest, S-phase arrest, G2/M arrest, spindle checkpoint arrest, and M-phase arrest.

In G1/S arrest, cyclin D recruits CDK4 or CDK6 to form the cyclin D-CDK4/6 complex, which phosphorylates pRB, leading the transcription factor E2F to be released from pRB and to activation of cyclin E transcription. Cyclin E combines with CDK2 to form a complex, further phosphorylating pRB in a positive feedback loop and enhancing S-phase activities, promoting the transition from G1 to S phase^203–206^. However, exposure to IR may contribute to interruption of the G1/S transition, resulting in S-phase arrest. In theory, G1/S arrest would give cells with radiation exposure more time to perform DNA damage repair^207–209^. Previous studies have indicated that p53, a famous transcription factor, regulates the cell cycle^210,211^, especially by monitoring G1 and G2/M checkpoints^195^. G1 arrest has been reported to be associated with p53 status. Nagasawa et al.^212^ revealed the absence of G1/S arrest in cancer cells expressing normal p53 synchronized by mitotic selection following irradiation. Fabbro et al.^213^ found that p53 was phosphorylated and regulated by a series of proteins. First, BRCA1 is phosphorylated at two sites, Ser1423 and Ser1524, based on the regulation of ATM/ATR (ATM and Rad3-related), and then, ATM/ATR is activated by phosphorylation of BRCA1 to phosphorylate p53 at the Ser15 site. Consequently, phosphorylation of p53 serves to monitor G1/S arrest by inducing p21, which is reported to be a CDKi. Yoon et al.^214^ compared the G1 population difference post IR between colon cells with p53 (+/+) or without p53 (−/−) expression, and the results illustrated that the G1 population was significantly abolished in p53 (−/−) cancer cells compared with that in p53 (+/+) cancer cells post IR. They also found that KLF4 mediated p53 activation to control G1/S arrest following irradiation, indicating that p53 regulation in the IR response in cancer cells is complex and that p53 is a key factor in the process. Thus, recovery or activation of p53 could be a strategy for overcoming RR. Jiang and Wang^215^ showed that downregulation of mitochondrial transcription factor A increased the radiotherapy sensitivity of cancer cells through the p53 signaling pathway. It has now been confirmed that when DNA damage occurs in G1 cells, the G1/S checkpoint can be triggered via at least two signaling pathways, the ATM/p53/p21 and the ATM/CHK2/CDC25C pathways^216^. For instance, post irradiation, ATM is activated, and ATM phosphorylates p53 and MDM2, promoting dissociation of p53 from MDM2 and inhibiting p53 translocation from the nucleus to cytoplasm; on the other hand, CHK2 is activated, which phosphorylates and stabilizes p53, and the increased level of p53 triggers the transcription of downstream genes such as *p21*, contributing to G1/S arrest^217–219^. Compared to the ATM/p53/p21 pathway, the ATM/CHK2/CDC25C pathway induces rapid signaling in response to DNA damage^220^. G1/S arrest is induced when CDC25C is degraded via ATM/CHK2 after IR-induced DNA damage ^221–223^.

During the IR-induced cell injury response, S-phase arrest is activated to inhibit DNA synthesis^224^. Various patterns, including DSBs, DNA crosslinks, and DNA adducts, can trigger S-phase arrest^195,225^. Deficiencies in S-phase checkpoints accelerate DNA synthesis under the condition of DSBs, an abnormal phenomenon that can occur in cells from patients with ataxia–telangiectasia (A–T), Nijmegen breakage syndrome, or other chromosome syndromes^225,226^. According to previous studies, ATM mediates S-phase checkpoints via three parallel signaling pathways: ATM/CHK2/CDC25a/CDK2, ATM/MRN/SMC1, and ATM/MRN/RPA^225,227^. In addition, post IR, ATM phosphorylates both Nbs1 and CHK2, leading to S-phase checkpoint activation. Ultimately, the distinct steps of DNA replication are suppressed^227^. Regulation of the S-phase checkpoint is complex, involving multiple pathways; thus, determining whether cancer cells are dependent on one, both, or neither of these intra-S-phase checkpoints in response to IR is necessary.

**Fig. 5:**
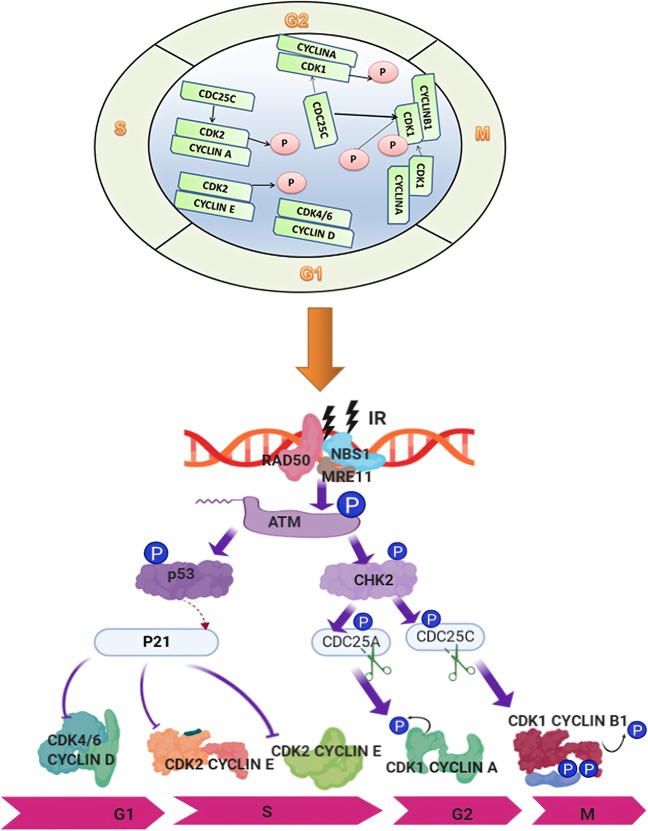
Functional complexes of cyclins and cyclin-dependent kinases (CDKs) and the signaling pathways involved in the regulation of cell cycle checkpoints in response to IR-induced DNA damage. CDK4/6/cyclin D promotes progression through the G1 phase. In late G1, the active CDK2/cyclin E complex promotes the G1/S transition. The CDK2/cyclin A complex promotes progression through S phase. The CDK1/cyclin A complex regulates progression through the G2 phase in preparation for mitosis. The G2/M-phase transition is initiated and promoted by the CDK1/cyclin B complex. The activity of CDK1/cyclin B is tightly maintained by the CDC25C phosphatase. Following DSB induction by IR, ATM is activated by the MRN complex, which then phosphorylates p53. Activated p53 transactivates the expression of p21^Cip1^, which inhibits CDK2, consequently inducing G1/S arrest. On the other hand, ATM phosphorylates and activates CHK2, which in turn phosphorylates and inactivates CDC25C; the latter is then cytoplasmically sequestered by 14-3-3 proteins. Consequently, the inhibitory phosphorylation of CDK1 by Wee1 and Myt1 on Tyr15 and Thr14 is maintained, and G2/M arrest is induced

G2/M arrest prevents cells from entering the M (mitosis) phase in the presence of DSBs^224^. G2/M arrest often occurs at 0.5 to 4 h post exposure to IR in mammalian cells and then resolves^228^. The higher the IR dose, the more obvious the G2/M arrest, and the more delayed the recovery effect; sometimes recovery is impossible, and cell death occurs as a result of mechanisms such as mitotic catastrophe. However, it should be noted that the level of cell cycle arrest and recovery time differ in different cell lines. In addition, a deficiency in some genes involved in regulating G2/M arrest, including PLK1, ATM, and CHK1, alters the cell cycle response to IR-induced DNA damage^229,230^. G2/M arrest is also associated with RR. Gogineni et al.^231^ found that when meningioma cells were subjected to IR, G2/M arrest could be triggered quickly, and the key event in the underlying mechanism was the phosphorylation of CHK2, CDC25C, and CDC2; this phosphorylation interferes with CHK2 activation and the cyclin B1/CDC2 interaction, resulting in permanent arrest followed by apoptosis. Aninditha et al.^232^ compared the effects of heavy ions and photons on malignant melanoma cell G2/M arrest and found that heavy ions caused a greater increase in G2/M arrest than photons, showing that heavy ions have better properties for improving RR for malignant melanoma cells than photons. A study conducted by Wang et al.^233^ showed that IR increased colorectal cancer cell sensitivity to the melatonin by triggering G2/M arrest as well as downregulating the expression of ATM, a key mediator in DSB repair. Peng et al.^228^ showed that in response to IR, radioresistant cells exhibited a recoverable G2/M phase during a prolonged cell cycle. These data validate the potential of targeting G2/M-related proteins involved in the response to IR to control RR in cancer patients. Figure 5 illustrates the regulation of cell cycle checkpoint-related proteins in the response to IR.

## Targeting DNA damage repair to sensitize cancer cells to radiation

DSBs generated by radiotherapy are the most efficient molecular events damaging and killing cancer cells; however, the inherent DNA damage repair efficiency of cancer cells may cause cellular resistance and weaken the therapeutic outcome. Genes and proteins involved in DSB repair are targets for cancer therapy since their alteration, interaction, translocation, and regulation can impact the repair process, making cancer cells more resistant or more sensitive to radiotherapy. Thus, targeting DNA damage repair as a method to sensitize cancer cells to radiotherapy is a promising therapeutic strategy for the precise and effective treatment of cancer patients. In this section, recently reported literature regarding some of the most important DDR-associated proteins involved in RR is reviewed and discussed.

### Targeting DNA-PKcs

DNA-dependent protein kinase (DNA-PK) is first introduced in this section due to its importance and because it has been intensively studied previously in the NHEJ pathway.^234^. It has been confirmed that DNA-PKcs can identify DSBs post-IR insult and facilitate “messy” broken end processing and DNA ligation by recruiting the proteins responsible for DNA damage repair processing and ultimately ligating the broken DNA ends^235,236^. DNA-PKcs was first discovered due to the observation that dsDNA can modulate the phosphorylation of a series of proteins^237^. In early published reports, DNA-PKcs was associated with repairing DSBs through the NHEJ pathway; however, with further study, it was illustrated that DNA-PKcs has multiple functions, including selection of NHEJ and HR repair pathways^238–241^, regulation of cell cycle checkpoints^242–245^, and maintenance of telomeres^245–247^. As one of the largest family members of the PIKK (phosphatidylinositol 3-kinase-related kinase) family, DNA-PKcs consists of 4128 amino acids^248^. During the past few decades, extensive studies have been conducted to reveal how DNA-PKcs works in DDR pathways^71,249^. Briefly, DNA-PKcs and other proteins, including Artemis and XLF, are recruited by Ku to form a DNA-PK functional complex^250^. Then, DNA-PKcs phosphorylates components of the NHEJ machinery, and autophosphorylation or ATM-catalyzed phosphorylation at Thr2609, Ser2056, and Thr2647 allows for DNA end processing^251–253^. Cells harboring decreased levels of DNA-PKcs showed increased sensitivity to IR compared to control cells^254^. In the HR pathway, replication protein A coupled with the phosphorylation of p53 affected HR in a mechanism mediated by DNA-PKcs ^255^.

Our research team has focused on studying DNA-PKcs for almost three decades. We have reported multiple essential DNA-PKcs functions and mechanisms in the IR-induced DDR. For instance, cyclin B1 ubiquitination was activated and its protein stability was affected by DNA-PKcs via the CDH1-APC pathway^256^. We also found that radioresistance may be a result of the effect of c-Myc on ATM phosphorylation and DNA-PKcs kinase activity^257^. Furthermore, DNA-PKcs associates with PLK1 and contributes to chromosome segregation and cytokinesis^242^. We demonstrated the effects of anti-DPK3-scFv on radiosensitization by targeting DNA-PKcs^258^. Another discovery was that Ku could recruit DNA-PKcs and CHK2^245^, and a dominant role for DNA-PKcs in regulating H2AX phosphorylation in the post-IR-induced DDR was identified^259^. Moreover, suppression of DNA-PKcs changes multiple signal transduction-associated genes at the transcriptional level and eventually affects cell proliferation and differentiation^260^. Our studies further identified that DNA-PKcs is a critical component of DNA damage repair pathways.

To date, DNA-PKcs is the best known regulator/mediator of the IR-induced DDR; furthermore, it has been implicated as an emerging intervention target in cancer therapy, particularly in radiotherapy or genotoxic chemotherapy^261^. More recently, the targeting of DNA-PKcs has been used in cancer radiotherapy. According to Liu et al.^262^, suppression of DNA-PKcs was markedly abrogated by the IR-induced transcription factor hypoxia-inducible factor-1α (HIF-1α), leading to IR-induced decreases in migration and invasion and enhanced radiotherapy sensitivity in glioblastoma. Mamo et al.^263^ reported that inhibition of DNA-PKcs sensitized human osteosarcoma cells in response to IR. Targeting DNA-PKcs with various inhibitors has been reported to be effective for potentiating radiotherapy and has been proposed as an effective strategy to improve cancer patient outcomes^264^. In recent decades, significant progress has been made in the development of large amounts of DNA-PKcs inhibitors from basic experiments, and many treatments have already been tested in clinical trials or applied in clinical therapy. Wortmannin was the first identified DNA-PKcs inhibitor^265^, but it lacks specificity, and its in vivo toxicity makes it difficult to use in clinical applications. Another nonselective inhibitor is LY294002, which has a similar structure to wortmannin^266^. Davidson et al.^266^ summarized a series of DNA-PKcs inhibitors in their review, which included LY294002, NU7026, NU7441, IC86621, IC87102, IC87361, OK-1035, SU11752, vanillin, NK314, and IC486241. LY294002 is a competitive inhibitor that binds reversibly to the kinase domain of DNA-PK^267^. Compared to nonselective inhibitors, NU7026 is more selective for DNA-PKcs^268^. NU7441 is a strong inhibitor of DNA-PKcs. It could improve RR in liver cancer cells by participating in DDR pathways and activating cell cycle arrest^269^. IC86621, IC87102, and IC87361 are inhibitors based on the LY294002 structure. These inhibitors promoted increased sensitization to IR and decreased repair of spontaneous and IR-induced DSBs^270^. Vanillin, derived from vanilla pods, showed selective inhibition of DNA-PK and specifically affects NHEJ^271^. In our laboratory, we identified a vanillin derivative, BVAN08, as a DNA-PKcs inhibitor that can efficiently induce autophagic cell death and mitotic catastrophe in radioresistant cancer cells. In addition to DNA repair inhibition, cancer cell killing by BVAN08 is related to destruction of the c-Myc oncoprotein and G2/M-phase function^272,273^. In addition to these inhibitors, several novel inhibitors have been recently discovered and published. M3814, an oral DNA-PK inhibitor, showed preclinical activity^274^. Sun et al.^275^ demonstrated that M3814 effectively blocked IR-induced DSB repair. AZD7648 is reported to be a potent and selective DNA-PKcs inhibitor, enhancing radiation sensitivity^276^. VX-984, a novel drug that was developed as a selective inhibitor of DNA-PKcs, enhanced cell death during radiotherapy^277^. Doxycycline, the first US Food and Drug Administration (FDA)-approved DNA-PK inhibitor, reduced DNA-PKcs protein expression by ~15-fold and functioned as a radiosensitizer in breast cancer cells^278^. A recent study showed that phosphorylation of H2AX and KAP1 could be facilitated by DNA-PKcs, resulting in chromatin decondensation and quickly recruiting the DDR complex to DNA damage sites ^279^.

### Targeting ATM/ATR

ATM was discovered during a clinical case observation; that is, in 1967, Gotoff et al.^280^ reported that a patient with a rare inherited autosomal-recessive genetic A–T condition exhibited immunodeficiency. A previous study showed that patients with A–T were more sensitive to radiotherapy than patients without A–T^281^. Subsequently, a study indicated that not only G1/S arrest but also G2/M arrest could not be activated in A–T cells after IR exposure^282^. Later work identified the *ATM* gene, with a transcript size of 12 kb^283^. ATR, originally discovered in budding yeast, has been found to exhibit S and G2 checkpoint deficiency^284^. Later work identified the C-terminal phosphoinositide 3-kinase (PI3K)-like kinase domain while cloning the *MEC1* gene because the sequence was similar to Mec1/Esr/Sad3 and fission yeast Rad3^285^. In 1996, Bentley et al.^286^ identified that ATR could functionally enhance esr1-1 radiation sensitivity in *Saccharomyces cerevisiae*. With the discoveries of ATM and ATR, multiple lines of scientific enquiry converged.

Both ATM and ATR are large polypeptides, and their domain organizations are similar, but their structural features are generally different^126^. One of the main roles of ATM and ATR is that they can phosphorylate serine or threonine residues according to some biochemical reports^287^. ATM and ATR share certain substrates and have some overlapping functions. ATM is activated and recruited to DSB sites by the MRN complex, which serves as a DNA damage sensor, while ATR is activated and recruited to DSB sites with its stable binding partner ATRIP (ATR-interacting protein)^288^. Previous studies have demonstrated that ATM is a master regulator of the cellular response to DSBs^289^. Indeed, the major ATM function is that it can initiate a cascade for the DSB signaling response, resulting in the phosphorylation of almost hundreds of substrates when cells are undergoing the DDR^290^. For instance, ATM activates CHK2 kinase and phosphorylates multiple sites, triggering apoptosis, and cell cycle arrest, and deficiency of ATM-mediated signaling reactions causes sensitization of cells to IR^291^. A recent study demonstrated that resting peripheral blood lymphocytes were more sensitive to radiotherapy when ATM was inhibited than when it functioned normally. Meanwhile, it was found post IR that ATM phosphorylation activity was decreased through stimulation of CD3/CD28^292^. Moreover, the synergistic relationships among three key proteins, ATM, ATR, and DNA-PKcs, have been found to present different functions with low- or high-dose IR. When subjected to low-dose IR, the G2 checkpoint is tightly regulated by ATM and ATR mainly through interactions with another cell cycle mediator, CHK1. However, when subjected to high-dose IR, the ATM and ATR complex becomes relaxed. Both ATM and ATR can affect the G2 checkpoint independently, resulting in DSB end resection^293^. Thus, some experts, such as Blackford and Jackson^126^, suggest that ATM, ATR, and DNA-PKcs react with each other, forming a complex and mediating DDR.

As key mediators of the DDR, the ATM and ATR kinases have been suggested to have extreme potential for improving radiotherapy outcomes because of their abilities to promote DDR and mediate cell cycle arrest^294^. To date, reported ATM inhibitors include caffeine, wortmannin, CP-466722, KU-55933, KU-60019, and KU-559403. Caffeine was first reported to sensitize cells to IR in 1995, and an increased radiosensitizing effect was observed in cells with p53 deficiency^295^. Wortmannin targets both ATM and DNA-PKcs to increase cell radiosensitivity. KU-55933 is potentially a selective ATM inhibitor. It confers marked sensitization to IR^296^. KU-60019 is an analog of KU-55933, inhibiting ATM downstream signaling and sensitizing cells to IR in vitro^297^. Glioblastoma-initiating cell-driven cancers with low p53 expression and high PI3K expression might be effectively radiosensitized by KU-60019^298^. Reported ATR inhibitors include schisandrin B, NU6027, NVP-BEZ235, VE-821, VE-822, AZ20, and AZD6738. Schisandrin B, reported in 2009, is a natural extract from the medicinal herb *Schisandra chinensis*. In human lung cancer cells treated with ultraviolet light, it could inhibit the activity of ATR phosphorylation substrates and abrogate G2/M cell cycle checkpoints^299^. NU6027, a nonselective inhibitor, has been shown in a few cancer cell lines to have potential for improving IR-induced RR. NVP-BEZ235, another reported potential and effective inhibitor, caused marked radiosensitivity in Ras-overexpressing cancers. VE-821 has been shown to be a potent ATR inhibitor by inhibiting phosphorylation of the ATR downstream target CHK1 at Ser345 in the colorectal cancer cell line HCT116. As a key inhibitor, VE-822 has been found to have potential because of its ability to increase persistent DNA damage. VE-822 was also reported to decrease HR for cancer cells post IR^300^, suggesting that this inhibitor is promising for overcoming RR in patients with advanced pancreatic ductal adenocarcinoma. AZ20 is another inhibitor of ATR^301^. A phase I study of AZD6738 was conducted to analyze the tolerability, safety, and biological effects of palliative radiotherapy in cancer patients in the United Kingdom in 2019^302^. These ATM and ATR inhibitors were identified based on a broad range of preclinical studies and extensive literature; however, the potential for increased normal tissue toxicity is likely to be an important concern, and identifying their selective effects in concert with radiotherapy will require further investigation.

### Targeting DNA LIG4

DNA LIG4 is an essential DNA repair component in the radiation-induced NHEJ pathway^303^. Commonly, LIG4, XRCC4, and Cernunos-XLF are recruited to the breakage site and temporarily attach to the ends of the DNA to ensure ligation of the DSB^304^. LIG4 deficiency leads to a rare primary immunodeficiency called LIG4 syndrome^305^. Patients who have been diagnosed with LIG4 syndrome present increased sensitivity to radiotherapy, but they also have an increased risk of neurological abnormalities and bone marrow failure, as well as increased susceptibility to cancer^306^. The symptoms of LIG4 syndrome show that LIG4 is vital in the DDR. Numerous studies have reported that mutations in LIG4 confer clinical radiosensitization. Riballo et al.^307^ indicated that mutation of LIG4 impaired the formation of an adenylate complex in addition to reducing the rejoining activity. Furthermore, healthy participants with the rs1805388 polymorphism of LIG4 were more sensitive to radiation based on γH2AX foci analysis than healthy participants without this polymorphism^308^. Nevertheless, a Lig4^−/−^p53^−/−^ cell line had a higher sensitivity to high-LET radiation than a Lig4^+/+^p53^−/−^ cell line, suggesting that LET-induced DNA damage is partially repaired through LIG4^309^ McKay et al.^310^ screened tissues from a unique bank of samples from radiosensitive cancer patients for expression defects in major DSB proteins such as LIG4. LIG4 and RCC4 proteins showed reduced expression in addition to a corresponding reduction in both gene products at the mRNA level. The impact of LIG4 on RR may be due to the following reported molecular mechanisms. The activity of LIG4 is regulated by other proteins, such as XRCC4, which is the key contributor to the stabilization of LIG4^311^. In addition, DNA-binding protein-1 negatively regulates DNA repair processes by downregulating the expression of LIG4^312^. Indeed, a LIG4 peptide was shown to be a substrate of DNA-PK in vitro; a phosphorylation site for DNA-PKcs is present at Thr650 in human LIG4, and LIG stability is regulated by multiple factors, including negative regulation by DNA-PK ^313^.

Screening for inhibitors of LIG4 offers a chance for target-based drug discovery to design RR drugs. Tseng et al.^314^ conducted a screen of 5280 compounds and found that rabeprazole and U73122 could specifically block the adenylate transfer step and DNA rejoining to inhibit IR-induced DNA damage repair by targeting LIG4. SCR7, identified by Srivastava et al.^315^, blocks LIG4-mediated joining by interfering with its DNA binding. NU7026 affects the radiosensitivity of wild-type LIG4 mouse embryonic fibroblasts^316^. Although inhibitors of LIG4 are considered potential anticancer drugs, they are likely not effective in cancer cells with mutations, which may affect radiotherapy outcomes. Thus, in the future, more investigations regarding LIG4 functions in the IR-induced DDR need to be conducted.

### Targeting PARP-1

PARP-1 is the most extensively studied nuclear enzyme of the PARP superfamily^317^. As reported previously, PARP-1 has been suggested to be a key regulator of DNA damage repair^318^. In the response to DNA damage, poly (ADP-ribosyl)ation (PARylation) of proteins is an initial reaction^319^. For instance, in the case of DNA damage, PARP-1 recognizes NAD^+^ as a major substrate^320^. PARylation of proteins post translation is suggested to provide a local signal of DNA damage because of the existence of poly(ADP-ribose)-binding domains, and DDR factors can regulate the functions of relevant proteins^321^. Furthermore, PARP-1 has been extensively studied, as it is a widely known regulator of DNA damage repair, particularly DSB repair ^322^.

Mechanistically, PARylation targets include nuclear DDR proteins, such as DNA-PKcs^323^ and PARP-1 itself. Since PARylated proteins can associate with negatively charged PAR, PARylated proteins can interact with DNA as well as regulate DNA damage as a signal^324,325^. Moreover, recent evidence has shown that PARP-1 has the potential for catalyzing the heterodimer formed with XPC-RAD23B and free PAR, suggesting the critical role of PARP-1 in IR-induced DNA damage ^319^.

Based on research indicating that inhibition of PARP-1 might sensitize cancer cells to radiotherapy^326,327^, the function of PARP-1 in DNA repair has been utilized in targeted radiotherapy. Currently, targeted radionuclide therapy with PARP-1 is a novel approach for cancer therapy^328^. Jannetti et al.^329^ reported that ^131^I-labeled PARP-1 therapy showed high potential for treating mice with glioblastoma, as the mice showed significantly longer survival than mice that received control vehicle in a subcutaneous model. Inhibitors of PARP-1 have also been developed to enhance the sensitivity of cancer cells to radiotherapy. The first identified inhibitor was nicotinamide, ~30 years ago^330^. After that, several generations of PARP-1 inhibitors were identified. Many of these inhibitors have been shown to enhance the anticancer efficacy of DNA-damaging agents such as IR^331,332^. For inhibitors of PARP (PARPi), their structures are similar to nicotinamide. PARPi mainly perform the following two functions. One is to inhibit PARP-1 catalytic activity, and the other is restrict PARP-1. The aims are typically to either inhibit PARylation or suppress PARP-1 release^333,334^. However, some older PARP-1 inhibitors showed limitations, such as nonselectivity and nonspecificity, in clinical radiotherapy^331^. Recently, some more specific and effective novel inhibitors have been developed. Ryu et al.^335^ suggested that the PARP-1 inhibitor KJ-28d might enhance the sensitivity of NSCLC to radiotherapy. Olaparib was the first PARP inhibitor approved (in December 2014) for cancer therapy by the FDA (https://www.astrazeneca.com/media-center/press-releases/2014/lynparza-approved-us-fda-brca-mutated-ovarian-cancer-treatment-19122014.html#) and by the European Union (https://www.astrazeneca.com/media-center/press-releases/2014/lynparza-approved-european-union-brca-mutated-ovarian-cancer-treatment-18122014.html#). It was approved for use in patients with advanced ovarian cancer and BRCA mutations. In January 2016, the US FDA further granted the Breakthrough Therapy Designation to olaparib for treating patients with metastatic-castration-resistant prostate cancer carrying BRCA1/2 or ATM mutations (https://www.astrazeneca.com/media-center/press-releases/2016/Lynparza-Olaparib-granted-Breakthrough-Therapy-Designation-by-US-FDA-for-treatment-of-BRCA1-2-or-ATM-gene-mutated-metastatic-Castration-Resistant-Prostate-Cancer-28012016.html#). Generally, cancer cells lacking either of the tumor suppressors BRCA1 and BRCA2, which are key components in the HR pathway of DSB repair, are selectively sensitive to PARP family inhibitors. Mechanistically, SSBs are primarily repaired by PARP-1. However, when DNA damage is caused by PARPi, inhibiting PARP-1 may not be lethal because there are still other repair pathways that function in the DDR, such as HR. However, the absence of BRCA1/2 results in a deficiency of HR activity, and cytotoxicity is present because DNA lesions caused by PARPi cannot be repaired due to the lack of HR activation^336^. Bourton et al.^337^ demonstrated that compared to normal BRCA cells, BRCA1^+/−^ lymphoblastoid cells treated with olaparib, followed by IR exhibited decreased BRCA1 protein levels and increased apoptosis, resulting in radiation hypersensitivity; these results suggest that the combination of a PARP-1 inhibitor with radiotherapy has clinical relevance in treating BRCA1-associated cancers. AZD2281 is also an effective radiosensitizer for carbon-ion radiotherapy, indicating that PARP-1 has a wide therapeutic range in combination with LET radiation by blocking the DNA damage repair response^327^. The PARP-1 inhibitor ABT-888 enhanced radiosensitizing effects in hepatocellular carcinoma^338^. In cell experiments, Mk-4827, a PARP-1/2 inhibitor, promoted lung and breast cancer cell sensitivity to radiation^339^. Some inhibitors have been implicated to improve therapy sensitivity or inhibit cancer recurrence in cancer patients in the clinic. For example, niraparib is used in patients with ovarian cancer^340^. Niraparib is also found to inhibit the DDR in cancer cells, leading to an initial sensitization of cancer cells to radiotherapy^341^. Collectively, although PARP-1 inhibitors have been identified and tested after clinical trials and their function in enhancing the response of cancers to IR has been documented, the underlying mechanisms of radiotherapy sensitization by these inhibitors remain to be fully elucidated ^342^.

### Targeting HIF-1

The 2019 Nobel Prize in Physiology or Medicine was awarded to three scientists, William Kaelin, Gregg Semenza, and Peter Ratcliffe, for their work on the discovery of HIF-1 as the gene switch controlling the cellular identification of and response to changed oxygen status^343^. HIF-1 is formed by HIF-1α and HIF-1β^344^, belonging to the basic helix–loop–helix PER-ARNT‐SIM (bHLH‐PAS) protein family^345^. HIF-1α becomes stable in response to hypoxia, but is degraded under normal conditions, while HIF-1β is constitutively expressed. Since HIF-1α expression changes under hypoxic conditions, most studies have focused on the effect of HIF-1α on the cellular identification of and response to oxygen levels. There are multiple functional domains in HIF-1α; in particular, the bHLH and PAS domains are linked to dimerization, DNA binding, and oxygen-dependent degradation domains that make HIF-1α more susceptible to proteasomal degradation under normal conditions and transactivation domains that are responsible for the activation of HIF-1 target genes^346,347^. HIF-1 activation mainly occurs due to hypoxia caused by vascular damage under radiotherapy conditions^348^, as well as due to reactive oxygen species (ROS) produced from IR insult^349^. Moreover, IR exposure upregulates glucose availability, promoting HIF-1α translation^350^. In radioresistant NSCLC cells, PAI-1 was secreted post IR through upregulation of HIF-1α, leading to the radioresistance of adjacent cells in a paracrine manner^351^. In addition, increased ROS production stabilizes HIF-1α, enhancing cancer-related fibroblasts through glycolysis, leading to the Warburg effect and the secretion of lactate to feed nearby cancer cells, ultimately inducing RR^352^. As metabolic changes occur in radioresistant cancer cells, several signaling pathways are activated by IR with altered HIF-1 expression^353^. Presently, investigation of the metabolic mechanisms is ongoing. HIF-1 signaling can also be activated by IR through damage to endothelial cells, producing hypoxia and regulating VEGF (vascular endothelial growth factor) and CXCL12 (C-X-C motif chemokine 12)^354^. HIF-1 is involved in the mitochondria-mediated RR of cancer stem cells^355^. In fact, HIF-1 has the potential to improve RR and increase radiotherapy outcomes.

In 2007, in a review by Brown^356^, it was noted that in cancer therapy, it is important to consider that cancer cells exist under a wide range of oxygen concentrations, which include those concentrations induced by hypoxia and necrosis, and one therapeutic strategy is to selectively induce HIF-1 under hypoxic condition. To this end, a series of HIF-1 inhibitors have been developed in the past decade. Typically, HIF-1-associated inhibitors can be divided into the following categories: adamantyl-based inhibitors, boron-based inhibitors, sulfonamides, moracins, manassantin A and its analogs, chalcones, ring-truncated deguelin derivatives, YC-1-related derivatives, and heterocycles. Among these reported inhibitors, some have been associated with RR. LW6 improved resistance to radiotherapy in hypoxic lung cancer cells by inhibiting the accumulation of HIF-1α^357^ via inhibition of the mitochondrial respiratory chain. Another HIF-1 inhibitor, PX-478, could enhance pancreatic cancer cell sensitivity to radiotherapy. The potential mechanism may be related to inhibiting cancer cell proliferation and, in particular, activating HIF-1 proangiogenic signaling to reverse RR in hypoxic cancer cells^358^. YC-1, another potential HIF-1 inhibitor, could reverse RR in A549 lung cancer cells by changing the optical redox (OR) status of cancer cells^358^. Topotecan was found to alleviate IR-induced brain necrosis in a mouse model^359^. However, most HIF-1 inhibitors are nonselective and do not directly inhibit HIF-1 protein. Furthermore, the HIF-1 signaling pathway is very complex. More than 1% of genes present high sensitivity to hypoxia. Thus, in future studies, we need to address the following questions: What are the underlying mechanisms of potential selective HIF-1 inhibitors? How do they influence RR: Notably, it should be mentioned that the effects of most HIF-1 inhibitors may be off-target, indicating that other signaling pathways may also be affected by these inhibitors. Hence, during the discovery process of novel selective inhibitors, knowledge of the molecular mechanism of action is essential.

### Targeting HDACs

One of the roles of histone deacetylases (HDACs) is to remove acetyl groups from the amino-terminal lysine residues by catalyzing reactions on histone proteins^360^. HDAC expression is broad, but the highest expression is mainly in the brain, heart, muscle, and testis. According to investigations of the role of HDACs in cancer RR, HDACs act as guardians of IR-induced DNA damage and promote RR^361^. For instance, HDAC6 knockdown predisposes glioblastoma cancer cells to radiotherapy-induced apoptosis ^361^.

Most HDAC inhibitors (HDACis) were discovered recently. They can target HDACs, improving RR. Various biological processes can be affected by these inhibitors. Cell growth, apoptosis, DNA repair, and terminal differentiation are the main processes influenced. HDACis have been found to suppress many proteins important in the DDR by downregulating proteins in the HR and NHEJ repair pathways in vitro^362^. HDAC induces the acetylation of histone and nonhistone proteins and modulates the acetylation of proteins related to DSB repair^363^. Several HDACis have been approved through clinical trials. They are short-chain fatty acids, benzamides, hydroxamic acids, and cyclic tetrapeptides^364^. Their approval information can be reviewed on clinical trial websites such as https://www.who.int/ictrp/ and https://clinicaltrials.gov/. Their molecular mechanisms involve enhancing radiation exposure by targeting multiple RR pathway molecules, including EGFR (epidermal growth factor receptor) and AKT^365,366^. Furthermore, HDACis overcome RR through physical modification of chromatin structure. HDACis may also acetylate heat-shock protein 90 (HSP90), promoting receptor degradation. Most importantly, HDACis are involved in DSB repair through prolonged expression of γH2AX and therefore downmodulate RAD51 and DNA-PK expression, eventually sensitizing cancer cells to IR^367,368^. In the past decade, several novel HDACis have been studied in cancer cell and animal models. Wang et al.^369^ reported that curcumin-mediated HDAC inhibition suppressed the radiation-induced DDR and led to elevated DNA damage sensitivity, indicating that curcumin is important for DSB repair. A study by Frame et al.^370^ demonstrated that trichostatin A conferred radiosensitivity to prostate stem-like cells. In addition, suberoylanilide hydroxamic acid (SAHA) enhanced radiotherapy sensitivity and suppressed lung metastasis in breast cancer, indicating that SAHA may serve as a potential sensitizer for radiotherapy^371^. Robert et al.^363^ found that HDACis, such as trichostatin A, resulted in both physical and functional alteration of PARP-1 binding at DSBs, potentially avoiding NHEJ processes in cancer cells and eventually decreasing NHEJ repair.

### Targeting CDK1

CDK1 plays an essential role in cell cycle regulation through modulation of the centrosome cycle and mitotic onset, enhancement of the G2/M transition, and regulation of G1 progression and the G1/S transition in combination with different cyclins^372^. CDK1 activity can be switched off by WEE1- or PKMYT1-mediated phosphorylation^373^. Once DNA damage occurs by IR, CDK1 is inactivated to stop the cell cycle at the G2 checkpoint to facilitate DSB repair^374^. CDK1 activation occurs by a series of steps. In brief, it first binds to cyclin B, and then, its phosphorylation of Thr161 is induced by the CAK/CDK7 complex; at the same time, its phosphorylation on Thr14 and Tyr15 sites is inhibited by CDC25, after which the CDK1/cyclin B complex is activated to redirect cyclin-kinase complexes from the cytoplasm to the nucleus^375^. In fact, because cancer cells usually promote CDK1 activity, this property can be useful for targeted cancer therapy^376^. Indeed, cells in S phase at the time of radiation exhibit a slowing of DNA synthesis mediated by the ATM/NBS1 and ATM/CHK2/CDC25A/CDK2 pathways^377,378^. Thus, the regulatory proteins that control cell cycle processes through CDK1 inhibition may be beneficial in radiotherapy.

Raghavan et al.^379^ tested a new-generation CDK1 inhibitor, AZD5438, in combination with radiotherapy in lung cancer lines, such as A549, H1299, and H460. The results showed that AZD5438 significantly enhanced the radiation sensitivity of lung cancer cells. RO-3306, a CDK1-targeting inhibitor, could promote activation of Bax and induce mitochondrial apoptosis, indicating that inhibiting CDK1 may be effective in acute myeloid leukemia cells^380^ by enhancing the downstream p53 signaling pathway. Yang and co-workers^381^ investigated the potential of taurine as an inhibitor of CDK1 and demonstrated that taurine significantly inhibited the IR-induced downregulation of CDK1. Notably, JNJ-7706621, an inhibitor that can inhibit both CDK1 and AURKA/B kinase concomitantly, has been shown to be able to revert radioimmunotherapy resistance in lymphoma cancer cells. This has an intriguing clinical implication: in some TP53-deficient cancers, the potential application of dual protein inhibitors may be a strategy to overcome IR-induced resistance. MEK162 is a nonspecific CDK1 inhibitor that downregulates and dephosphorylates multiple cell cycle checkpoint proteins, including CDK1, CDK2, and Wee1^382^, leading to a prolonged DNA damage signal in response to IR exposure in glioblastoma cells. In fact, regulation of the cell cycle in cancer cells is complex, and CDK1 can be upregulated or downregulated by multiple cell cycle checkpoint proteins. The regulation of cell cycle proteins in response to IR exposure can be thought to resemble a spider web. Exposure to IR is similar to the moth flying into the spider web; multiple proteins located in this web react immediately, regulating the interaction and mediating the integrity of the web. Thus, compounds that inhibit CDK1-related proteins may also inhibit CDK1 activity. Satyanarayana et al.^383^ showed that p21, which is upregulated by CDK1, is activated in response to γ-irradiation, resulting in the inhibition of CDK1 for further DNA damage repair. As cancer cells require specific interphase CDKs for proliferation, CDK1 inhibitors may provide a therapeutic benefit against RR.

### Targeting Wee1-like protein kinase (Wee1)

Wee1 serves as a negative regulator of cell cycle entry into mitosis at the G2 to M transition. Wee1 could mediate the phosphorylation of CDK1 at Tyr15. Wee1 could prevent the cyclin B1/CDK1 complex from relocating to the nucleus from the cytoplasm prior to the onset of mitosis^384,385^. Indeed, Wee1 phosphorylates and inactivates the cyclin B1/CDK1 complex in a specific way^386^. Wee1 activity increases during S and G2 and decreases in M phase when it is hyperphosphorylated^387–390^. Inhibition of Wee1 activity has been considered a potential avenue for cancer radiotherapy^391^.

To date, several Wee1 inhibitors have been investigated, some of which are concomitant CDK1 inhibitors, suggesting that Weel is an effective target for sensitizing various types of cancers to radiotherapy^392–394^. Experimental evidence has demonstrated that Wee1 inhibitor AZD1775 significantly inhibits cancer growth and impairs RAD51 focus formation in response to radiotherapy^394^. The mechanisms underlying how inhibition of the Wee1 activity by AZD1775, resulting in promotion of the G2/M transition, is to be considered^395^. In this context, cancer cells, which harbor G1 checkpoint aberrations, could enter premature mitosis; ultimately, mitotic catastrophe would occur because of this unsuccessful DDR^388^. To date, on the clinical study website (https://clinicaltrials.gov/), there are ~50 clinical studies regarding AZD1775, of which 12 have been completed, 16 trials are recruiting, and 9 have been terminated or withdrawn. Most of these clinical trials have been conducted in various cancers and feature combinations with chemotherapy or radiotherapy. MK 1775, a selective WEE1 inhibitor, sensitizes lung cancer cells to RIDD and apoptosis through suppression of Sirt1^396^. Human leukemic T cells pretreated with 681641, a Wee1 kinase II inhibitor, have significantly increased sensitivity to radiation. If pretreated with both inhibitors, 681641 and R1-1, a Rad51 inhibitor, further enhanced apoptosis was seen in cancer cells^397^. PD0166285, another reported WEE1 inhibitor, is a major drug for inducing radiotherapy sensitivity because it can lead cancer cells to fail to perform an IR-induced DDR^398^. Because of the important role of Wee1 in the G2/M checkpoint along with CHK1/2, using Wee1 inhibitors such as AZD1775 or MK 1775, can relieve G2 arrest, sensitizing cancer cells to radiotherapy.

### Targeting serine/threonine-protein kinase (CHK1)

Similar to Wee1 and CDK1, CHK1 is also required for checkpoint-mediated cell cycle arrest and activation of DNA repair in response to DNA damage. The mechanisms of CHK1 regulation are complex and involve multiple steps^399^. Briefly, during HR, CHK1 is activated by its interaction with RAD51, promoting the intra-S and G2/M cell cycle checkpoints and modulating the cellular response to replication stress^400^. Increasing evidence illustrates that cancer cell survival and proliferation can be targeted as potential strategies for sensitizing cells to radiotherapy because cell survival and proliferation are significantly affected by CHK1^401^. In 1996, UCN-01 became the first reported CHK1 inhibitor; it has broad-spectrum efficacy against the protein kinase C family^402^, but due to its nonspecificity and long half-life, its clinical usage has been limited. According to the clinical trials website (https://clinicaltrials.gov), 12 clinical trials on CHK1 inhibitors have been completed, including trials for LY2606368, PF-00477736, and SRA737. In the trial data, these CHK1 inhibitors showed promising anticancer combination effects with other drugs that could generate replication-dependent DNA damage^403,404^. LY2606368 was the first selective CHK1 inhibitor. King et al.^405^ reported that inhibition of CHK1 by LY2603618 contributed to reduced DNA synthesis and elevated H2AX phosphorylation, indicative of DNA damage and premature entry into mitosis. PF-00477736, a selective inhibitor, was evaluated for its potential for radiosensitization in several cancer cell lines. The results showed that PF-00477736 contributes to substantial radiosensitization^406^. SRA737, another inhibitor, was also found to have the potential to suppress cell growth when used in combination with niraparib^407^. However, some CHK1 inhibitors developed at an early stage have been found to induce serious adverse events; for example, a clinical study showed that 14.29% of participants treated with SCH900776, a CHK1 inhibitor, were at risk of cardiac disorders (https://clinicaltrials.gov/). During the past decade, several new inhibitors have been discovered. In 2017, Suzuki et al.^408^ reported that a novel CHK1 inhibitor, MK8776, promoted IR-induced cell death via enhancement of aberrant mitosis and exacerbated mitotic catastrophe at a minimally toxic concentration without influencing DNA damage repair. Another novel CHK1 inhibitor, CCT244747, the first orally bioavailable CHK1 inhibitor, sensitized bladder and head and neck cancer cells to IR through modulation of G2/M checkpoint control, suggesting that CCT244747 may be suitable for oral administration^409,410^. Notably, combined treatment of the CHK1 inhibitor AZD6738 with the WEE1 inhibitor AZD1775 exerted a significant synergistic cytotoxic effect in mantle cell lymphoma and diffuse large B cell lymphoma cancer cells with a marked S-phase delay and increased DNA damage, indicating that combination treatment may provide a promising therapeutic avenue for B cell lymphoma patients^411^. Another study also indicated that serious adverse cytotoxic effects were found when cancer cells were treated with three different targeted inhibitors, LY2606368, cisplatin, and talazoparib, together^412^. However, although some inhibitors alone or in combination with other inhibitors may induce certain adverse effects, these inhibitors show great potential for cancer therapy. Therefore, during the development of inhibitors, seeking low cytotoxicity and high efficacy agents targeting CHK1 is encouraged. The combination of CHK1 inhibitors with inhibitors of other targets may translate into effective clinical applications in the future.

Indeed, increasing our knowledge of the underlying mechanisms of IR-induced DDR as well as clarifying the precise roles of key genes and proteins and their interaction partners involved in DDR signaling pathways is critical to the clinical discovery of novel intervention targets and will aid in the eventual development of effective strategies against cancer. In fact, numerous studies have focused on DNA damage repair genes and proteins and their regulation, suggesting essential roles in sensitization to radiotherapy. These observations provide insights for more radiotherapy sensitization approaches to kill cancer cells selectively and specifically.

**Fig. 6:**
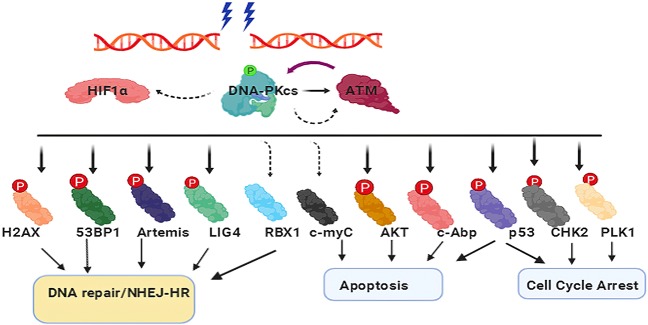
Interregulation of the PIKK family members DNA-PKcs and ATM and their downstream substrates in the DDR pathway activated following DSB induction by IR. The dotted arrow represents the regulation at the transcription level. The solid arrow indicates the kinase activity-dependent regulation at the post-translational level

Previous investigations of targets and inhibitors involved in radiotherapy-induced DNA damage repair have improved radiotherapy treatments targeting signaling pathways for various cancers. In the future, given the roles of these targeted signaling pathways associated with the IR-induced DDR, the relevant molecular mechanisms will require further elucidation to develop novel effective inhibitors and to decrease the toxicity of currently available inhibitors. Several current inhibitors have not achieved success in clinical trials, indicating the need for a better understanding of how these inhibitors participate in radiation-mediated DNA damage. Table 2 lists the targeted proteins involved in the response to radiotherapy. Table 3 lists the inhibitors of these targeted proteins and their functions in radiotherapy. Figure 6 displays the major downstream targets of ATM- and DNA-PK-mediated signaling pathways and their involvement in DDR.

**Table 2:** Targetable proteins involved in DNA damage repair in response to radiation

	Length	Activity regulation	Molecular function	Main biological processes^a^
DNA-PKcs	4128	Autophosphorylation or phosphorylation by ATM	ATP binding, DNA-dependent protein kinase activity, double-stranded DNA binding, protein domain-specific binding	DSB repair, negative regulation of the response to γ-radiation
ATM	3056	ATM dimers are normally inactivated but are activated with DNA in the presence of MRN; inhibited by wortmannin	ATP binding, DNA binding, DNA-dependent protein kinase activity, identical protein binding, protein serine/threonine kinase activity	Cell cycle arrest, cellular response to γ-radiation and X-ray radiation, mitotic telomere clustering, DSB repair via HR and NHEJ, DNA replication, DNA damage checkpoint
ATR	2644	Stimulated by TOPBP1^468^, activated by ETAA^469^, inhibited by caffeine, wortmannin, and LY294002	ATP binding, DNA binding, MutSα and MutLα complex binding, protein kinase activity	Cellular response to γ-radiation and UV light, DNA damage checkpoint, DNA repair, positive regulation of DDR, signal transduction, regulation of signal transduction by p53 class mediator, negative regulation of DNA replication
CHK1	476	Activated through phosphorylation	ATP binding, histone kinase activity, kinase activity, protein domain-specific binding, protein kinase activity	Cell cycle arrest, cellular response to γ-radiation and X-ray radiation, mitotic telomere clustering, DSB repair via HR and NHEJ, DNA replication, DNA damage checkpoint
LIG4	911	A direct target of β-catenin	ATP binding, DNA binding, DNA ligase activity, DNA ligase activity, metal ion binding, protein C terminus binding	Cell cycle, cell division, cell proliferation, cellular response to IR, DNA biosynthetic process, DNA ligation, DNA ligation involved in DNA repair, DSB repair via HR and NHEJ, response to γ-radiation and X-ray radiation, single-strand break repair
PARP-1	1014	NAD^+^ as a substrate and automodification of PARP‐1 itself^470^	DNA binding, enzyme binding, estrogen receptor binding, histone deacetylase binding, NAD binding, protein kinase binding	Apoptotic process, cellular response to DNA damage-stimulated DNA repair, DSB by HE, mitochondrial DNA repair, response to γ-radiation
WEE1	646	Activated through phosphorylation^471^	ATP binding, kinase activity, protein kinase activity, protein tyrosine kinase activity	Cell division, establishment of cell polarity, G2/M transition of the cell cycle, negative regulation of G1/S transition in the cell cycle
CDK1	297	Activated through phosphorylation	ATP binding, chromatin binding, cyclin binding, histone kinase activity, HSP70 protein kinase activity, protein serine/threonine kinase activity	Apoptotic process, mitotic G2 DNA damage checkpoint, positive regulation of G2/M transition in the cell cycle and gene expression and protein import into the nucleus, protein phosphorylation

^a^Available from https://www.uniprot.org/

**Table 3:** DDR inhibitors and their association with radiotherapy resistance

Target	Inhibitor	Cancer type	Result	Mechanism	Refs.
DNA-PKcs	VX-984	Glioblastomas	Concentration-dependent inhibition of radiation-induced DNA-PKcs phosphorylation	VX-984-induced radiosensitization is mediated by inhibition of DNA repair	^472^
	NU5455	Human orthotopic lung cancer	Enhanced the activity of doxorubicin	N/A	^264^
	KU60648	Osteosarcoma	Enhanced cell cycle distribution and DNA damage	Increased cell accumulation at the G2/M transition point and increased percentage of cells with γH2AX foci	^263^
	NU7441	Cervical and breast cancers	Enhanced radiosensitivity	Lowered cancer cell survival rates, increased apoptosis, and a G2-phase arrest	^473^
	NU7026	Non-small-cell lung cancer	Enhanced radiosensitivity and increased the levels of ATM and ATR	Promoted apoptosis and G2/M arrest	^474^
	Wortmannin	Bladder cancer	Enhanced radiation-induced apoptosis with defective p53 activity (bladder cancer cell line, RT112 cells)	Inhibited DNA-PK, resulting in the inhibition of DSB repair	^475^
ATM	AZD1390	Glioma	Resulted in tumor cell hypersensitivity to IR	Induced G2 cell cycle phase accumulation, micronuclei, and apoptosis	^476^
	AZ32	Glioma	Blocked the DDR	Interacted with mutant p53	^477^
	GSK635416A	Head and neck squamous cell carcinoma	Enhanced radiosensitization with cisplatin and cetuximab	Increased DSBs	^478^
	KU-55933	Bladder cancer	Enhanced radiosensitivity	Elevated ATM expression and S-phase cell distribution and DSB repair kinetics	^296^
ATR	AZD6738	Kras-mutant cancer	Attenuated radiation-induced CD8^+^ T cell exhaustion and potentiated CD8^+^ T cell activity.	Suppressed PD-L1 upregulation on tumor cells and decreased the number of tumor-infiltrating Tregs	^479^
	VE-821	HeLa cells	Enhanced radiosensitivity	Abrogated the G2/M checkpoint	^480^
HIF-1	BAY 87-2243	Non-small-cell lung cancer	Enhanced radiosensitivity	Inhibited mitochondrial complex I activity	^344,481,482^
	Saikosaponin-d	Hepatoma	Enhanced radiosensitivity	Upregulated p53 and Bax, downregulated Bcl-2 by attenuating HIF-1α expression under hypoxic condition	^483^
	BAY-84-7296	Squamous cell carcinomas	Enhanced radiosensitivity	N/A	^484^
HDAC	Trichostatin A (TSA)	Human cervical carcinoma	Enhanced radiosensitivity	Downregulated the expression of HIF-1α and VEGF proteins	^485^
	Vorinostat/SAHA		Enhanced radiosensitivity	Stimulated caspase activation consistent with apoptotic cell death	^486^
	ITF2357	Glioma	Enhanced radiosensitivity	Induced G1/S growth arrest	^487^
	LBH589	Undifferentiated pleomorphic sarcoma	Suppressed tumor growth	Induced apoptosis and G2/M cell cycle arrest	
WEE1	AZD1775	Hepatocellular carcinoma	Enhanced radiosensitivity	Delayed resolution of γH2AX foci and the induction of pan-nuclear γH2AX staining	^488^
	MK 1775	Lung cancer	Enhanced radiosensitivity	Suppressed Cdk1 phosphorylation	^489^
	PD0166285	Osteosarcoma (OS)	Enhanced radiosensitivity	Abrogated G2 arrest	^398^
CDK1	AZD5438	Lung cancer	Enhanced radiosensitivity	Inhibited Cdk1, prolonged G2/M arrest	^379^
CHK1	CCT244747	Bladder and head and neck cancer	Enhanced radiosensitivity	Abrogated G2 arrest	^409^
	MK8776	Human triple-negative breast cancer	Enhanced radiosensitivity	Suppressed autophagy	^490^

## Challenges for IR-induced signal transduction and targeted therapy

As discussed above and in previous studies, radiation induces genomic DNA damage, challenging the integrity of the genome, particularly by inducing DSBs and causing various types of chromosomal aberrations in cancer cells^413^. From bacterial to mammalian cells, DNA damage signaling kinases play a central role in coordinating the DDR^414^. Once exposed to IR, DNA damage signaling kinases mediate hallmark responses, including cell cycle arrest, initiation of apoptosis, and induction of transcription^415,416^. In addition, these DNA damage signaling kinases are also regulated in an integrated network. In their review, Lanz et al.^414^ noted that DNA damage signaling kinases target dozens, if not hundreds, of DNA repair proteins, thereby modulating repair pathways. Indeed, the DNA damage signaling kinases and their targets are regulated by a complex network, the underlying mechanisms of which remain to be completely elucidated. Below, we list a number of key scientific issues for a holistic view of the IR-induced DNA damage signal transduction network that may be of concern over the next decade:

1. As described above, phosphorylation events mediated by DNA damage signaling kinases in response to IR are critical for the control of DNA damage repair pathways. However, the substrates and underlying molecular mechanisms remain incompletely clear. For instance, inhibition of ATM and DNA-PKcs strongly impairs the NHEJ pathway, but we do not know how the previous phosphorylation events occur in this process or which key signal transductions are essential for NHEJ. With the development of techniques such as mass spectrometry and gene sequencing technology, the ability to quantitatively detect phosphorylation events in the process of IR-induced DNA damage repair can provide new insight for the identification of signaling kinase substrates and molecular mechanisms. Phosphoproteomics has been used not only to identify the targets of DNA damage signaling kinases in mammalian cell lines but also to identify phosphorylation events catalyzed by DNA damage signaling kinases^417–419^. Looking ahead, given the large datasets arising from mass spectrometry and high-throughput gene sequencing, statistical analysis methods must be developed for more efficient prediction and analysis.
2. How do DNA damage signaling kinases function differently when subjected to high-, low-, and very low-dose IR exposure? Similarly, at what dose of radiation do phenotypic alterations in DNA occur? While DDR and repair signaling pathways under conditions of high-dose IR exposure, such as the levels provided by clinical radiotherapy, have been intensively investigated, the effects of low-dose radiation, particularly environmental radiation exposure, such as that generated from pediatric computed tomography or chest examination by X-ray, on the DNA damage repair signaling pathways have been ignored. Kim et al.^420^ reported on how exposure to low-dose radiation affects the human body, showing that low-dose radiation stress causes DNA damage and induces DNA damage-related signaling pathways, including apoptosis, cell cycle arrest, and DNA repair. Nagle et al.^421^ reported that low-dose irradiation of high-density murine and human glandular stem cells represented a dose threshold for DNA damage repair activation, leading to low-dose hyperradiosensitivity. Gaetani et al.^422^ investigated the effects of low-dose IR on DNA damage in circulating cells of occupationally exposed workers; the results showed that among workers with low-dose IR exposure, DNA repair activity was increased, and moreover, workers with cancer cases in their family history showed significantly reduced 8-oxoguanine glycosylase 1-dependent DNA repair activity compared with those workers without any family history of cancer. Although the effects of low-dose radiation on DNA have attracted increasing attention over the past decade, the molecular mechanisms, biomarkers, and possible clinical applicability require further investigation.
3. What is the crosstalk between DSB repair pathways in response to radiotherapy? Current studies have focused on the roles of HR and NHEJ, the two main pathways involved in regulating IR-induced DSB repair, and cell cycle checkpoints. However, there may be other unidentified repair pathways that have yet to be discovered, and furthermore, as different repair pathways exist, there may also be crosstalk. Limpose et al.^423^ showed that base excision repair, which was suggested to function independently in a previous perspective, was proven to directly interact with other DDR pathways rather than operating in isolation. Sunada et al.^424^ demonstrated that breast cancer cells with BRCA1/2 mutation treated with a PARP inhibitor had improved susceptibility via the crosstalk of DSB repair pathways. Several novel regulatory mechanisms have been discovered using crosstalk assays. For instance, Yuan et al.^425^ conducted cell experiments and revealed a feedback loop of Yes-associated protein (YAP) and p53 protein, as well as SIRT1-mediated regulation of YAP-p53 feedback loop-related deacetylation in certain residues. The authors suggested that this regulatory route was a new mechanism related to lung tumorigenesis. Exploring the interplay between repair pathways is important for our understanding of fundamental processes relevant to improving radiotherapy outcomes. Currently, omics technology and high-throughput assays to simultaneously survey the crosstalk of multiple DNA repair pathways are being developed^69,426^. For instance, the CRISPR/Cas9 system has been widely used and is being improved rapidly.
4. Recently, increasing numbers of studies have shown that combining radiation therapy with immunotherapy triggers a series of cell responses, including inducing cell death. This area is drawing increasing attention in the clinical and scientific communities^427^. As a result, the identification of inhibitors with potential to improve radioimmunotherapy resistance should consider both the regulation of radiation-related signal transduction and immunotherapy signal transduction^428^. Furthermore, it is necessary to uncover the mechanisms underlying why some patients experience durable responses, while others develop therapy resistance when treated with the combination of radiotherapy and immune therapy. Importantly, a better mechanistic understanding of the combination of radiotherapy and immune therapy is needed to benefit more patients in clinical applications. It is worth considering whether and how the translocation of DNA fragments from the nucleus to the cytoplasm can be recognized and used to trigger a response that can be translated into a novel strategy of improving cancer cell killing at the cellular and whole body levels. It is well documented that the tumor microenvironment can be altered. For instance, post IR, some inflammatory cytokines and immune cells are altered significantly. Harding et al.^429^ revealed that cell cycle progression through mitosis following DSBs leads to the formation of micronuclei. Furthermore, the pattern-recognition receptor cyclic GMP-AMP synthase (cGAS) is a cytosolic DNA sensor that activates innate immunity prior to activation of inflammatory signaling. Low expression of cGAS in patients with lung cancer is associated with poor survival, likely because cGAS deletion abrogates the senescence-associated secretory phenotype^430^. Obviously, targeted therapies to activate the cGAS-STING pathway in cancer cells can mediate cellular senescence and activate antitumor immunity, which could be another promising strategy for providing significant therapeutic benefits for cancer patients.
5. Is enough data available to assess inhibitor toxicity, safety, and carcinogenicity for normal tissues? As discussed above, inhibition of DNA damage repair can improve radiotherapy sensitivity in a wide range of cancers. However, most inhibitors lack relevant toxicity assessments, safety assays, and investigations of the risk of carcinogenesis prior to clinical application. Although several studies have been conducted to indicate that inhibition of cyclooxygenase-2 or nuclear factor-κB sensitizes cancer cells without causing normal cell/tissue toxicity^431–434^, the underlying molecular mechanisms should be determined to improve our understanding of the toxicity. Likewise, it is also important to provide more selective, low toxicity, and higher efficiency radiotherapy treatments at a low cost for cancer patients. A newly published review by Pilie et al.^71^ pointed out that the current strategies for treating cancers target DDR signaling pathways; hence, when the current DNA damage repair inhibitors are under development or in clinical trials prior to use in the larger population, attention should be paid to minimizing overlapping toxicities.
6. Are there enough early and sensitive biomarkers for predicting, preventing, or controlling RR? Although current studies have provided multiple DNA damage sensors and DNA damage repair regulatory proteins as possible targets to improve radiotherapy sensitivity, the challenge ahead is how to translate basic radiobiology studies into clinical applications. Moreover, any innovation and progress in radiotherapy depend on insights realized by basic radiobiological research^435^.

Clinical applications are generated from the discoveries of radiobiological studies, including DNA damage repair signal transduction pathways and specific IR-induced DDR proteins, such as DNA-PKcs, ATM, and ATR. We would like to use a fairy tale to explain the key points of this review. RR is similar to a mermaid, whose scales can be considered similar to the different signaling transduction-related pathways and proteins. The mermaid wants to shed her scales and become human; however, without an understanding of from where each scale originates and how they are regulated by internal or environmental factors, the mermaid scales are difficult to remove. Once the underlying mechanisms have been elucidated, the mermaid can shed her scales and become a human being.

Indeed, studying signaling transduction pathways in the field of radiobiology will dramatically contribute to the development of targeted radiotherapies. More than one century of study in this field has borne plenty of fruit, and the quality of radiotherapy has gradually improved. Further studies should continue to uncover the underlying mechanisms of IR-induced DNA damage repair, cancer metabolism, cancer stem cells, and the cancer microenvironment, ensuring that our knowledge of radiobiology increases to improve the outcomes of cancer treatments.
